# Silica Coating of Ferromagnetic Iron Oxide Magnetic Nanoparticles Significantly Enhances Their Hyperthermia Performances for Efficiently Inducing Cancer Cells Death In Vitro

**DOI:** 10.3390/pharmaceutics13122026

**Published:** 2021-11-27

**Authors:** Cristian Iacoviță, Ionel Fizeșan, Stefan Nitica, Adrian Florea, Lucian Barbu-Tudoran, Roxana Dudric, Anca Pop, Nicoleta Vedeanu, Ovidiu Crisan, Romulus Tetean, Felicia Loghin, Constantin Mihai Lucaciu

**Affiliations:** 1 Department of Pharmaceutical Physics-Biophysics, Faculty of Pharmacy, “Iuliu Hatieganu” University of Medicine and Pharmacy, 6 Pasteur St., 400349 Cluj-Napoca, Romania; cristian.iacovita@umfcluj.ro (C.I.); stefan_nitica@yahoo.com (S.N.); nicoletavedeanu@yahoo.com (N.V.); 2 Department of Toxicology, Faculty of Pharmacy, “Iuliu Hațieganu” University of Medicine and Pharmacy, 6A Pasteur St., 400349 Cluj-Napoca, Romania; ionel.fizesan@umfcluj.ro (I.F.); anca.pop@umfcluj.ro (A.P.); floghin@umfcluj.ro (F.L.); 3 Department of Cell and Molecular Biology, Faculty of Medicine, “Iuliu Hatieganu” University of Medicine and Pharmacy, 6 Pasteur St., 400349 Cluj-Napoca, Romania; 4 Electron Microscopy Center “Prof. C. Craciun”, Faculty of Biology & Geology, “Babes-Bolyai” University, 5-7 Clinicilor St., 400006 Cluj-Napoca, Romania; lucian.barbu@ubbcluj.ro; 5 Electron Microscopy Integrated Laboratory, National Institute for Research and Development of Isotopic and Molecular Technologies, 67-103 Donath St., 400293 Cluj-Napoca, Romania; 6 Faculty of Physics, “Babes Bolyai” University, Kogalniceanu 1, 400084 Cluj-Napoca, Romania; roxana.dudric@ubbcluj.ro (R.D.); romulus.tetean@phys.ubbcluj.ro (R.T.); 7 Department of Organic Chemistry, “Iuliu Hațieganu” University of Medicine and Pharmacy, 41 Victor Babes St., 400012 Cluj-Napoca, Romania; ocrisan@umfcluj.ro

**Keywords:** iron oxide magnetic nanoparticles, silica coating, magnetic hyperthermia, cancer cells, alamar blue, neutral red, A549, A35, BJ

## Abstract

Increasing the biocompatibility, cellular uptake, and magnetic heating performance of ferromagnetic iron-oxide magnetic nanoparticles (F-MNPs) is clearly required to efficiently induce apoptosis of cancer cells by magnetic hyperthermia (MH). Thus, F-MNPs were coated with silica layers of different thicknesses via a reverse microemulsion method, and their morphological, structural, and magnetic properties were evaluated by multiple techniques. The presence of a SiO_2_ layer significantly increased the colloidal stability of F-MNPs, which also enhanced their heating performance in water with almost 1000 W/g_Fe_ as compared to bare F-MNPs. The silica-coated F-MNPs exhibited biocompatibility of up to 250 μg/cm^2^ as assessed by Alamar Blues and Neutral Red assays on two cancer cell lines and one normal cell line. The cancer cells were found to internalize a higher quantity of silica-coated F-MNPs, in large endosomes, dispersed in the cytoplasm or inside lysosomes, and hence were more sensitive to in vitro MH treatment compared to the normal ones. Cellular death of more than 50% of the malignant cells was reached starting at a dose of 31.25 μg/cm^2^ and an amplitude of alternating magnetic field of 30 kA/m at 355 kHz.

## 1. Introduction

Magnetic nanoparticles (MNPs) subjected to the action of an externally applied alternating magnetic field (AMF) generate heat, leading to an exciting new technique in cancer treatment, named magnetic hyperthermia (MH) [1,2,3,4]. As attested by different clinical trials on prostate carcinoma [5,6], glioblastoma [7,8], and breast malignant tumors [9], the MH offers the possibility of heating the targeted tumoral tissue while preserving the healthy tissue. In clinical trials, the MH treatment has been carried out using superparamagnetic iron oxide nanoparticles (SPIONs), which were previously approved for clinical use by the US Food and Drug Administration [10]. Due to the limited efficiency of heat generation by SPIONs in the local tumor tissues for achieving a high performance in cancer therapy, very high dosages of SPIONs have been used. Moreover, to facilitate the complete elimination of the tumor, the MH treatment with SPIONs has been used in conjunction with chemotherapy and radiotherapy, which are prone to aggressive side effects. Therefore, for clinical safety, the MH treatment requires MNPs to display high heating efficiency to reduce the applied dose and minimize the risk of side effects.

MNPs dissipate the magnetic energy of interaction with alternating magnetic fields (AMF) in the environment through hysteresis losses [11]. The heating behavior of SPIONs can be simply described in the frame of the so-called Linear Response Theory [12]. In this model, the magnetization of the SPIONs depends linearly on the external AMF and the heat generated and its dependence on the MNPs’ properties are explained in the terms of the Neel and Brown magnetic relaxations processes [11,12]. The cell internalization of SPIONs leads to an enhancement of intracellular clustering inside endosomes or lysosomes and inhibits their mobility in cells, restricting their physical rotation, and thus affecting the Brown relaxation process [13,14]. Overall, the specific absorption rate (SAR, the rate at which heat is dissipated by the MNPs) is dramatically reduced [15,16]. The replacement of SPIONs with larger MNPs in the ferromagnetic domain (F-MNPs) represents an alternative to improve the effectiveness of MH treatment in cancer therapy. The SAR values increase almost one order of magnitude in comparison with SPIONs, due to the increase in the size and to an increase in the dynamic hysteresis area resulting in enhanced hyperthermic efficacy [11,16,17,18,19,20,21,22,23,24]. Besides favorable MH performance, the F-MNPs have a significant drawback as magnetic dipole-dipole interactions among them are very strong and hinder the complete dispersion in solution. In this regard, several strategies of coating the surface of F-MNPs have been formulated aiming at improving their colloidal stability and to provide, at the same time, a high level of biocompatibility [25,26,27,28].

Silica coating represents a well-known technique for MNP surface modification concerning the main required features for their biomedical use [29,30]. For instance, the silica shell protects the magnetic core from corrosion and/or oxidation, maintaining its magnetic properties [31,32]; makes the MNPs hydrophilic and provides colloidal stability in biological solutions by avoiding interparticle interactions and agglomeration [33,34]; presents excellent biocompatibility, exhibiting no cell cytotoxicity at concentrations up to 500 μg/mL [35,36,37,38,39]; prevents iron release [40]; and generates high surface area available for further attachment of anticancer drugs or other biologically active moieties [41,42,43,44,45]. In terms of MH properties, individual MNPs coated with a silica shell, which protects them from aggregation when subjected to AMF, showed better heating performances in comparison with uncoated MNPs or with multiple MNPs encapsulated in a silica shell [35,37,38,39,41,46,47]. The excellent biocompatibility properties offered by the silica shell [35,36,37,38,39,40,48,49] facilitate the internalization of a large quantity of silica-coated MNPs inside cells which upon MH treatment effectively leads to the eradication of tumor cells in vitro [35,37,38,39,41,46,47]. The formation of a silica shell around MNPs has been mainly realized by the so-called Stöber process where tetraethyl orthosilicate (TEOS) is hydrolyzed in ethanol under the addition of aqueous ammonia. However, the Stöber method, widely used for silica coating of both either hydrophilic or hydrophobic SPIONs and F-MNPs, offers poor control over the silica shell thickness and uniformity and leads mainly to encapsulation of multiple MNPs [31,32,39,40,47,49,50,51]. Alternatively, a fine-tuning of silica shell thickness around single magnetic cores is offered by the water-in-oil micro-emulsion method. This method uses a non-ionic surfactant that facilitates ammonia-mediated TEOS hydrolysis on the surface of MNPs dispersed in an organic solvent [34,35,36,38]. To the best of our knowledge, the reverse microemulsion method provides the best results on SPIONs, as they exhibit superior colloidal dispersion as compared to F-MNPs. Additionally, there is no extensive study on the evolution of MH properties of F-MNPs upon their coating with a silica shell, or on their cytotoxicity and in vitro MH performances.

Based on the above description, the aim of this work was twofold: the optimization of the ferromagnetic polyhedral iron oxide MNPs (Fe_3_O_4_) coating with a homogeneous silica shell to increase their colloidal stability in water and their SAR, and, second, the evaluation of their cytocompatibility and their MH capabilities to induce cancer cell apoptosis in vitro. Employing the reverse microemulsion method, we were able to coat the polyhedral Fe_3_O_4_ with a silica shell of variable thickness. The structural, magnetic, and hyperthermia properties were studied as a function of the coating thickness and compared to the uncoated polyhedral Fe_3_O_4_. The silica-coated polyhedral Fe_3_O_4_ (sFe_3_O_4_) providing the best heating performance were further selected to evaluate their cytotoxicity, cellular uptake, and in vitro MH performance. The interaction of sFe_3_O_4_ with two types of cancer cell lines—human pulmonary cancer cells (A549) and human melanoma cancer cells (A375)—and one normal cell line, human foreskin fibroblasts (BJ), was considered. The cytotoxicity and intracellular MH were assessed using two complementary assays: the neutral red (NR)-uptake assay, which is related mainly to lysosomal activity of the cells, and the Alamar Blue (AB) assay, which provides more general information related to the whole-cell metabolism. The uptake of sFe_3_O_4_ MNPs by cells upon 24 h incubation was monitored by using scanning and transmission electron microscopy (SEM and TEM). These two experimental techniques together with the cytocompatibility assays were extensively used to evaluate the cellular viability and intracellular damages, respectively, upon exposure of cells loaded with different amounts of sFe_3_O_4_ MNPs to AMF of different amplitudes (up to 65 kA/m) at a constant frequency of 355 kHz.

## 2. Materials and Methods

### 2.1. Synthesis

All the reagents used for the synthesis of MNPs and their coating with a silica layer were of analytical grade and were used without any further purification. The following products were employed: iron (III) chloride hexahydrate (FeCl_3_∙6H_2_O) (Carl-Roth, Karlsruhe, Germany, ≥98%), polyethylene glycol 200 (PEG 200) (Roth, ≥99%), and sodium acetate trihydrate (NaAc) (Roth, ≥99.5%), ethanol (Chemical, Iași, Romania, Chemicals, 99.9 %), cyclohexane (Sigma Aldrich, Steinheim, Germany), Igepal CO-520 (Sigma Aldrich, St. Louis, MI, USA), ammonium hydroxide (25%) (Chemicals), tetraethyl orthosilicate (TEOS) (Sigma Aldrich, ≥99%), and APTES (Sigma Aldrich, 99%).

The synthesis of MNPs was performed following a polyol mediated synthetic route as presented previously by our group [52,53]. Upon multiple washing steps, the MNPs were redispersed in ethanol at a concentration of 4 mg_MNPs_/mL. The MNPs were coated with silica via a reverse microemulsion method [35]. Briefly, 18 mL cyclohexane and 1.15 mL Igepal CO-520 were mixed for 30 min. Afterward, 4 mg of MNPs dispersed in 2 mL cyclohexane were added while stirring. After 5 min, 0.05 mL APTES and 0.05/0.1/0.2 mL TEOS were added, followed by 0.15 mL ammonium hydroxide (25%). The dispersions were stirred at room temperature for 24 h and the resulting MNPs-SiO_2_ were precipitated by adding ethanol. The collected MNPs were washed in ethanol several times and finally redispersed in water.

### 2.2. Characterisation

Transmission electron microscopy (TEM) images of MNPs were obtained using a Hitachi HD2700 (Hitachi, Tokyo, Japan) microscope operating at 200 kV, coupled with an EDX (energy-dispersive X-ray) detector (Oxford Instruments, AZtec Software, version 3.3, Oxford, UK) used for elemental detection. Samples were prepared by depositing a drop of MNP dispersion on a carbon-coated copper grid and removing the excess water by filter paper after 2 min.

X-ray diffraction (XRD) measurements were performed using a Bruker D8 Advance diffractometer using Cu Kα radiation (Bruker AXS GmbH, Karlsruhe, Germany). The powder samples were realized by collecting the MNPs with a magnet and vacuum drying them overnight. The FullProf software (FullProf.2k, Version 7.00-May 2019-ILL JRC https://www.ill.eu/sites/fullprof/, Grenoble, France) was employed to detect the crystalline phases and to calculate the lattice parameters.

Fourier transform infrared spectroscopy (FTIR) spectra were recorded between 400 and 4000 cm^−1^ at 4 cm^−1^ resolution using a TENSOR II instrument (Bruker Optics Inc., Billerica, MA, USA) in attenuated total reflectance mode using the platinum attenuated total reflectance (ATR) accessory with a single reflection diamond ATR. A few microliters of the aqueous solution of MNPs were allowed to dry on the diamond crystal, and the average spectrum of 16 scans was recorded for each sample.

Hydrodynamic size and zeta potential measurements of the MNPs were determined using a Zetasizer Nano ZS90 (Malvern Instruments, Worcestershire, UK) in a 90° configuration. Particle solutions with concentration of 0.1 and 0.01 mg_MNPs_/mL were used for measurements at room temperature. Each assay was made in triplicate.

The magnetic characterization of MNPs was carried out using a Cryogenic Limited (London, UK) vibrating sample magnetometer (VSM). The magnetization and hysteresis curves were measured from 0 to 10 T and −4 and 4 T, respectively, at both 4 and 300 K. The samples consisted of powder obtained in a similar way as for XRD examination.

The heating ability of MNPs was determined using a commercially available magnetic hyperthermia system, the Easy Heat 0224 from Ambrell (Scottsville, NY, USA) equipped with a fiber-optic thermometer. An AC magnetic field with fixed frequency (355 kHz) and variable amplitude (5–65 kA/m) was generated by an 8-turn coil that was made of a water-cooled copper tube. For measurements, 0.5 mL of MNPs suspended in water at different concentrations was placed in the center of the coil using a thermally isolated Teflon holder. Details about SAR calculation and iron concentration determination are provided in the Supplementary Materials.

### 2.3. Cell Lines

Two types of cancerous cancer cell lines (human pulmonary cancer cells—A549 and human melanoma cancer cells—A375) and one normal cell line (human foreskin fibroblasts—BJ) were used in the current study. All cells were maintained in Dulbecco’s Modified Eagle Medium (DMEM, Gibco, Paisley, UK) supplemented with 10% Fetal Bovine Serum (FBS, Sigma Aldrich, Steinheim, Germany). Cells were cultured in flasks at 37 °C in a humidified incubator with 5% CO_2_ supplementation while cellular media was changed every other day. At 70–80% confluence cells were subcultured or harvested for experiments.

### 2.4. In Vitro Cytocompatibility Assays

The cytocompatibility of NPs was evaluated on the cancerous and normal cell phenotypes by performing two complementary assays, namely Alamar Blue (AB) and Neutral Red (NR) assays. AB was used to measure the metabolic ability of viable cells to convert resazurin to resorufin, whereas the NR assay was used to measure the ATP content of exposed cells. In the case of both assays, 750,000 cells for A549 and A375 and 275,000 cells for BJ suspended in 2 mL of media were seeded in 6-well plates and left to attach for 24 h before MNP exposure. Cell concentration was selected to achieve a confluence of 70% before the exposure to the MNPs. Cells were exposed to 1000 µL of cell medium containing MNPs to reach a concentration of 500, 250, 125, 62.5, and 31.25 µg_MNPs_/cm^2^. After a 24 h incubation, the cells were washed three times with PBS (Gibco, Paisley, UK) and further incubated with the AB and NR dye, and dissolved in cellular media, for measuring the cellular viability. For the AB assay, cells were incubated for 3 h with 200 µM resazurin solution, and the fluorescence was measured at λ_excitation_ = 530/25 nm; λ_emission_ = 590/35 nm, using a Synergy 2 Multi-Mode Microplate Reader. For the NR assay, cells were incubated with a filtered neutral red dye (40 μg/mL) solution for 2 h, followed by a wash with PBS and the extraction of the accumulated cellular dye in a solution containing 50% ethanol, 49% water, and 1% glacial acetic acid. Supernatant fluorescence was measured at λ_excitation_ = 530/25 nm; λ_emission_ = 620/40 nm, using a Synergy 2 Multi-Mode Microplate Reader. The experiments were conducted on at least three biological replicates and included a negative control (cells exposed to culture medium). The results were normalized to the value of negative control (100%).

### 2.5. Evaluation of Cellular Uptake

The uptake of silica-coated MNPs in cells was quantitatively determined by the thiocyanate assay and qualitatively evaluated by SEM and TEM image analysis. In the case of both assays, the same number of cells as above suspended in 2 mL of media were seeded in 6-well plates and left to attach for 24 h. Afterward, the cells were exposed to 1000 µL of cell medium containing MNPs to reach a concentration of 62.5, 31.25, 15.62, and 7.81 µg_MNPs_/cm^2^. Upon 24 h incubation, the cells were washed 3 times with PBS to remove attached MNPs from the cellular surface.

For quantitative measurement of the intracellular Fe^3+^ using the thiocyanate assay, cells were trypsinized and centrifuged at 4500× *g* for 5 min, then further processed as described in Section S6.

SEM analysis of all three cell lines grown on glass coverslips (previously carefully cleaned and disinfected) was performed. At the end of incubation with silica-coated Fe_3_O_4_, culture media containing suspended nanoparticles were removed from the wells, and cells were washed 3 times with PBS to remove unbound MNPs. Cells were pre-fixed with 2.7% glutaraldehyde (Agar Scientific, Stansted, UK) solution in 0.1 M phosphate buffer (pH = 7.4) for 2 h and next washed 4 times with the same buffer. Fixation was performed at 4 °C with 1.5% OsO_4_ (Sigma-Aldrich, Steinheim, Germany) solution in 0.15 M phosphate buffer (pH = 7.4) for 1.5 h. Finally, an ethanol (VWR International, Fontenay-sous-Bois, France) series of increasing concentrations (50%-absolute—15 min each at room temperature) was used for dehydration. The coverslips fragments with the adherent cells were fastened on 10 mm/Ø9 mm aluminum stubs (Bio-Rad, Hercules, CA, USA), using carbon adhesive tabs (Electron Microscopy Sciences, Hatfield, PA, USA) and colloidal silver (Polaron Equipment, Watford, UK). Two samples were prepared for each group. They were next sputter-coated with gold in a Polaron E-5100 sputter coater (Polaron Equipment, Watford, UK). The examination was performed with a Jeol JSM 25-S (Jeol, Tokyo, Japan) at 30 kV, and relevant images were recorded with a Pixie 3000 system (Deben, Debenham, UK) and subjected both to a qualitative and quantitative evaluation. The general aspect of cells, presence, and the number of thick branches, and the number of filopodia were considered.

For TEM analysis, the cell suspensions were centrifuged for 5 min at 300 g and the pellets were further processed for TEM examination. Pre-fixation was performed for 2 h at 4 °C with 2.7% glutaraldehyde solution in 0.1 M phosphate buffer; the pellets were washed 4 times with the 0.1 M phosphate buffer at 4 °C; post-fixation was performed for 1.5 h with 1.5% OsO_4_ solution at 4 °C; for dehydration, an acetone (International Laboratory, Cluj-Napoca, Romania) series of increasing concentrations (30%-absolute—15 min each at room temperature) was used, and embedding was performed in three steps with EMBED 812 (Electron Microscopy Sciences, Hatfield, PA, USA) epoxy resin (the last two overnight) at room temperature. After polymerization (72 h at 60 °C), ultrathin sections were cut with a Diatome diamond knife (DiATOME, Hatfield, PA, USA) on an LKB Ultrotome III Bromma 8800 ultramicrotome (LKB Produckter AB, Stockholm-Bromma, Stockholm, Sweden), collected on 3 mm 300 mesh Cu grids (Agar) (covered with a formvar film—Electron Microscopy Sciences, Hatfield, PA, USA) and contrasted with 13% uranyl acetate (Merck, Darmstadt, Germany) for 7 min at room temperature. Examination of sections was performed with a Jeol JEM 1010 at 80 kV, and relevant images were recorded with a Mega View G2 camera (Olympus Soft Imaging Solutions, Münster, Germany). On the TEM images, the presence and location of MNPs within the cells were analyzed, in addition to the ultrastructural changes generated by the simple presence of the MNPs in the three types of cells, and by the in vitro induced magnetic hyperthermia.

### 2.6. In Vitro Magnetic Hyperthermia

A549, A375, and BJ cells were seeded in 6-well plates and exposed for 24 h to 62.5, 31.25, 15.62, and 7.81 µg/cm^2^ of MNPs. Cells were washed thoroughly with PBS, trypsinized, equally split into two aliquots of 1500 µL, and centrifuged for 10 min at 100× *g* to obtain a pellet. After the removal of 1300 µL of cell culture media, one aliquot was subjected to an AMF for 30 min, whereas the other aliquot maintained at 37 °C in a water bath served as a negative control. For the MH assays, three intensities of the AMF—30, 45, and 60 kA/m—were used, while the frequency was 355 kHz. Upon exposure to the AMF, cells were seeded in 96-well plates (6 technical replicates) and the viability was evaluated after 24 h using the AB and NR assays, described in Section 2.4. Cellular viability was expressed as the relative values between the viability of cells loaded with MNPs and exposed to AMF and negative control (cells exposed to MNPs but not to AMF). For the evaluation of cellular and intracellular damages induced by the MH treatment, biological samples were prepared for TEM and SEM analysis as described in Section 2.5. The experiments were performed with three biological replicates.

### 2.7. Statistics

The results are presented as mean values ± standard deviation (SD). Unless stated otherwise, the normally distributed data sets were analyzed using a one-way analysis of variance (ANOVA), and for quantitative assessment of cellular parameters Bonferroni multiple test comparison was also applied. Data analyses and graphical representation were performed in SigmaPlot 11.0 computer software (Systat Software Inc., Chicago, IL, USA), and GraphPad Prism 7.00 (GraphPad Software, San Diego, CA, USA). Results showing *p*-values less than 0.05 were considered statistically significant.

## 3. Results and Discussion

### 3.1. Structural Characterization of sFe_3_O_4_

**Figure 1:**
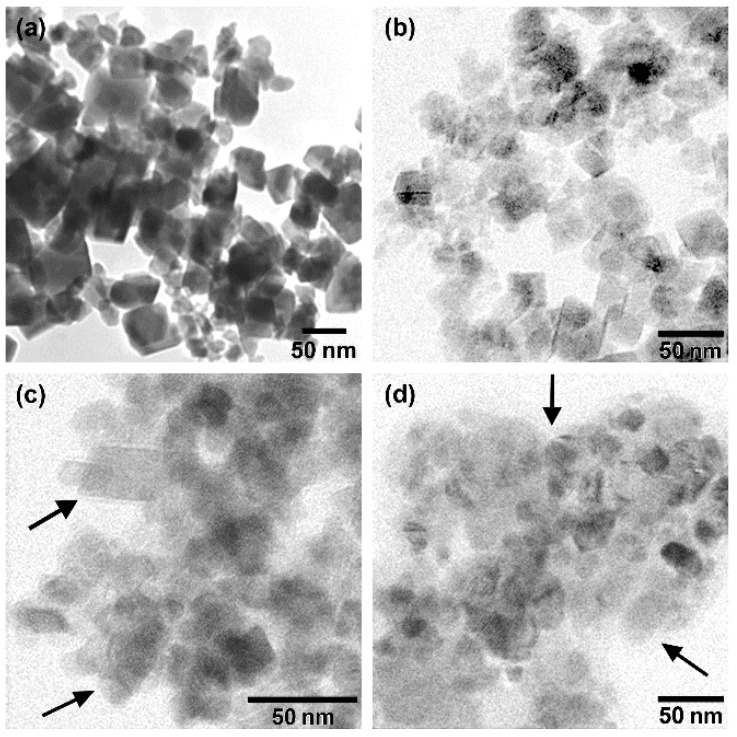
TEM images of (**a**) bare polyhedral Fe_3_O_4_ MNPs and sFe_3_O_4_ MNPs: (**b**) Fe_3_O_4_@SiO_2_-1, (**c**) Fe_3_O_4_@SiO_2_-2, and (**d**) Fe_3_O_4_@SiO_2_-3. The black arrows indicate the silica shell.

The preparation of silica-coated magnetite nanoparticles (sFe_3_O_4_) consisted of two steps. Firstly, ferromagnetic polyhedral Fe_3_O_4_ with an average length of 42 nm (Figure 1a) were prepared using a modified polyol synthesis method as described by us previously [52]. The as-synthesized polyhedral Fe_3_O_4_ MNPs were then coated with silica by employing the reverse microemulsion method [34,35,36,38]. The amount of TEOS used in the reaction mixture was varied, while the other components were kept constant, the resulting samples being denoted as Fe_3_O_4_@SiO_2_-1, Fe_3_O_4_@SiO_2_-2, and Fe_3_O_4_@SiO_2_-3. When using 50 μL of TEOS, the silica shell formed around Fe_3_O_4_ MNPs was very thin and therefore difficult to observe in TEM images (Figure 1b). By increasing the amount of TEOS at 100 μL in the reaction mixture, the presence of silica coating around Fe_3_O_4_ MNPs was easily seen in TEM images (black arrows in Figure 1c). The amorphous silica shell, surrounding the Fe_3_O_4_ exhibiting a dark contrast, is seen as a bright fringe. Thicker silica coating around Fe_3_O_4_ MNPs was achieved using 200 μL of TEOS in the silica coating process (black arrows in Figure 1d). The polyhedral morphology of Fe_3_O_4_ is similar in all three cases, indicating that it is not affected by the silica coating. Please note that the polyhedral Fe_3_O_4_ are ferromagnetic at room temperature; thus, once dispersed in different solvents, they are stabilized in aggregates of different dimensions as a function of the MNPs’ concentration [24]. Therefore, as compared to SPIONs, where the silica shell surrounding individual MNPs has a uniform thickness, in our case, the similar silica coating process implies the coating of assemblies of Fe_3_O_4_ MNPs (Figure 1 and Figure S1). For this reason, it is difficult to perform a precise quantification of the silica coating thickness. However, a closer look at both TEM (Figure 1) and STEM (Figure S1) images reveals that the contrast with sFe_3_O_4_ MNPs decreases and their profile became less and less visible, upon increasing the TEOS amount in the preparation method. These observations indicate that our approach, based on the variation of TEOS amount in the reverse microemulsion method contributed to the formation of sFe_3_O_4_ MNPs with variable silica coating thickness.

**Figure 2:**
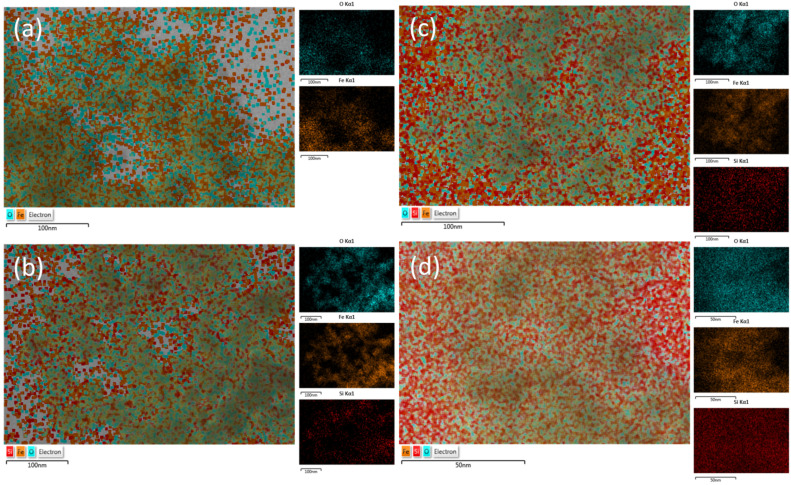
Elemental EDX mapping of (**a**) bare polyhedral Fe_3_O_4_ MNPs and sFe_3_O_4_ MNPs: (**b**) Fe_3_O_4_@SiO_2_-1, (**c**) Fe_3_O_4_@SiO_2_-2, and (**d**) Fe_3_O_4_@SiO_2_-3. Oxygen (blue), iron (orange), and silicon (red) atoms.

The chemical composition and the distribution of chemical elements within the sFe_3_O_4_ MNPs were evaluated by energy-dispersive X-ray (EDX) spectroscopy and mapping. Figure 2 shows the EDX mapping of oxygen (blue), iron (orange), and silicon (red) of the as-produced bare and silica-coated Fe_3_O_4_ MNPs. Both iron and oxygen atoms were found to be homogeneously distributed within the total volume of polyhedral MNPs (Figure 2a) in agreement with the XRD patterns. Furthermore, in the case of Fe_3_O_4_@SiO_2_-1 MNPs, the silicon atoms were mainly detected at the edges of polyhedral Fe_3_O_4_ MNPs and not mixed with iron atoms, which supports the formation of core/shell nanoparticles (Figure 2b). The red dots, representing the silicon atoms, appear more densely around polyhedral Fe_3_O_4_ MNPs together with oxygen atoms, indicating an increase in the silica shell (Figure 2c). For the last sample, Fe_3_O_4_@SiO_2_-3 MNPs, the silicon atoms dominate the EDX map, whereas the iron atoms are faintly visible (Figure 2d), which reveals the formation of thicker silica shell around polyhedral Fe_3_O_4_ MNPs.

As demonstrated in our previous work [53], the high-temperature polyol synthesis method produces faceted magnetic nanoparticles containing mainly the magnetite phase. In this sense, the Rietveld refinement of the XRD pattern matched that of magnetite (PDF number: 88-0315) and was consistent with the cubic Fd-3m crystal structure for magnetite with no other phases being evident within the estimated uncertainty of the measurement (which is not possible for a sample containing predominantly maghemite). Moreover, the corresponding lattice parameter of polyhedral NPs (a = 0.8388 nm) was very close to that of pure magnetite NPs (a = 0.83952 nm) as compared to pure maghemite NPs (a = 0.8364 nm). The saturation magnetization had a high-value characteristic of magnetite. XRD analysis was further employed to determine whether the silica coating process affects the crystallinity and purity of Fe_3_O_4_ MNPs. For the three samples containing sFe_3_O_4_ MNPs (Figure S2b–d), the position and the relative intensities of all diffraction peaks correspond to those ascribed to Fe_3_O_4_ MNPs magnetite (Figure S2a). The identical XRD patterns found for all four samples indicate that the silica-coated process did not affect the crystallinity of the magnetite core. Instead, the related peaks of sFe_3_O_4_ MNPs progressively broaden and decrease in intensity as the amorphous silica shell thickness is increased (Figure S2). A slight decrease in the corresponding lattice parameter was also recorded for sFe_3_O_4_ MNPs, but the values are very close to that of bulk magnetite (a = 0.8375 nm), suggesting that the purity of the magnetic core is retained upon silica coating.

### 3.2. FT-IR Spectroscopy and Colloidal Properties of sFe_3_O_4_

**Figure 3:**
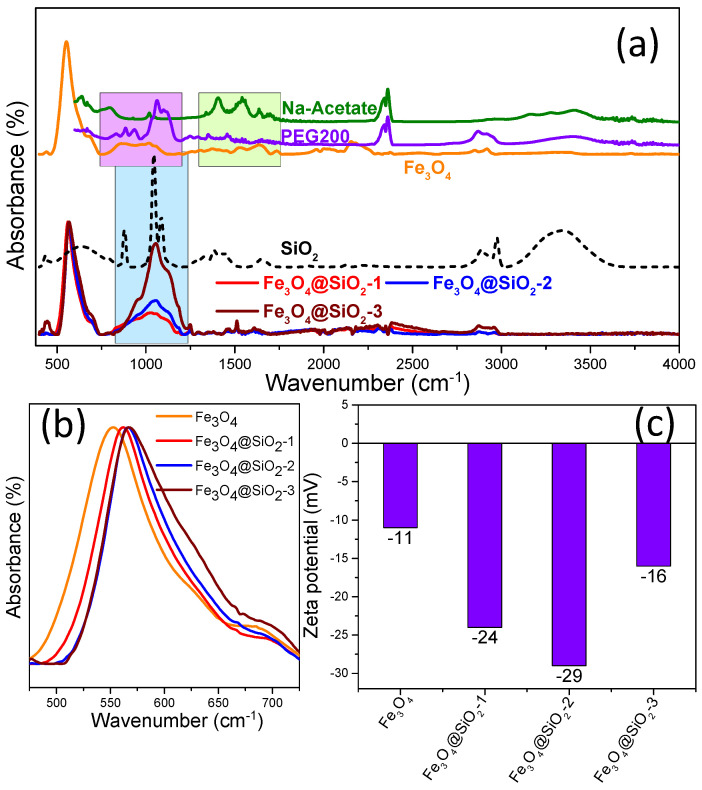
(**a**) FT-IR spectra of bare polyhedral Fe_3_O_4_ MNPs (orange), PEG200 (purple), Na-acetate (green), SiO_2_ (black) and sFe3O4 MNPs: Fe_3_O_4_@SiO2-1 (red), Fe_3_O_4_@SiO2-2 (blue) and Fe_3_O_4_@SiO2-3 (brown). The spectra are normalized to the highest absorption band and are vertically shifted for clarity. (**b**) Fe-O vibrational band of bare polyhedral Fe_3_O_4_ MNPs (orange), Fe_3_O_4_@SiO2-1 (red), Fe_3_O_4_@SiO2-2 (blue), and Fe_3_O_4_@SiO2-3 (brown) MNPs. (**c**) Zeta potential values of Fe_3_O_4_, Fe_3_O_4_@SiO_2_-1, Fe_3_O_4_@SiO_2_-2, and Fe_3_O_4_@SiO_2_-3 MNPs dispersed in water at 0.01 mgMNPs/mL.

In addition to the direct observation of the silica layer around Fe_3_O_4_ MNPs in TEM images and EDX mapping, FT-IR spectroscopy confirmed the silica coating on Fe_3_O_4_ MNPs. The FT-IR spectrum of uncoated Fe_3_O_4_ MNPs, dominated by the Fe–O absorption band around 540 cm^−1^, also reveals the characteristic absorption bands of PEG200 (in 750–1250 cm^−1^ region—purple rectangle) and those of the stretching vibrations of the carboxylate group of the Na-acetate (in 1300–1800 cm^−1^ region—green rectangle) (Figure 3a). Upon coating the polyhedral Fe_3_O_4_ MNPs with a silica shell, all vibration bands related to PEG200 and Na-acetate disappeared from the FT-IR spectra which exhibited the absorption band characteristics for SiO_2_ (blue rectangle): Si–O–Si stretching vibrations between 1000 and 1250 cm^−1^ were the most prominent; the shoulder at 965 cm^−1^ is attributed to Si–OH vibrations, whereas the small absorption band at 475 cm^−1^ reflects the Si–O–Si bending [34,35,40,54]. As compared to the dominant absorption band representing the Fe–O bond, the intensity of the SiO_2_ characteristics absorption bands increases when passing from Fe_3_O_4_@SiO_2_-1 to Fe_3_O_4_@SiO_2_@-2 and finally to Fe_3_O_4_@SiO_2_-3 (Figure 3a). This supports the TEM and EDX analysis, according to which the increase in TEOS amount in the coating procedure leads to increasing silica shell thickness around polyhedral Fe_3_O_4_ MNPs. This is also reflected in a progressive shift of the Fe–O vibrational band towards higher wavenumbers (Figure 3b).

The polyhedral Fe_3_O_4_ MNPs presented a negative value of the zeta potential of −11 mV (Figure 3c), most probably due to the presence of acetate onto their surface (Figure 3a). The coating of polyhedral Fe_3_O_4_ MNPs with a silica shell rendered the sFe_3_O_4_ MNPs more negative: the zeta potentials of Fe_3_O_4_@SiO_2_-1, Fe_3_O_4_@SiO_2_-2, and Fe_3_O_4_@SiO_2_-3 were −24, −29, and −16 mV, respectively (Figure 3c), in agreement with the results obtained in another study [40]. The hydrodynamic diameter of polyhedral Fe_3_O_4_ MNPs at a very low concentration of 0.01 mg_MNPs_/mL was about 280 nm (Figure S3a), which is much greater than the average size resulting from TEM images [53]. Due to the ferromagnetic state of polyhedral Fe_3_O_4_ MNPs at room temperature [53], it is obvious that they form clusters in the colloidal suspension, as also revealed by TEM images. For the case of sFe_3_O_4_ MNPs, the hydrodynamic diameter increased as the silica shell increased: 325 nm for Fe_3_O_4_@SiO_2_-1, 355 nm for Fe_3_O_4_@SiO_2_-2, and 465 nm for Fe_3_O_4_@SiO_2_-3 (Figure S3a). This highlights that the silica coating process involves clusters of MNPs to the detriment of individual MNPs.

The polydispersity index (PDI) for bare Fe_3_O_4_ MNPs is 0.27 (Table S1) which indicates a quasi-narrow size distribution of clusters. PDI decreases for Fe_3_O_4_@SiO_2_-1 and Fe_3_O_4_@SiO_2_-2 samples to 0.23 and 0.18, respectively. This suggests that the silica coating process involving up to 100 μL of TEOS reduces the size distribution of clusters. Instead, for the Fe_3_O_4_@SiO_2_-3, the PDI increases to 0.34, which suggests that a higher amount of TEOS in the reaction mixture enables the formation of thicker silica later around clusters, increasing their size distribution.

DLS measurements performed at a concentration of 0.1 mg_MNPs_/mL (ten times higher) revealed different colloidal behavior of the samples. For instance, the hydrodynamic diameter of both Fe_3_O_4_ and Fe_3_O_4_@SiO_2_-3 MNPs increased considerably to 1650 and 830 nm, respectively (Figure S3b). Taking into account that these two samples presented the smallest zeta potential values, it can be considered that the negative charges on the MNPs surface do not produce sufficient repulsive force to balance the magnetically induced attractive force. The latter interaction dominates as the concentration of MNPs increases in the colloidal suspension, causing the formation of aggregates with larger sizes. On the contrary, the more negatively charged Fe_3_O_4_@SiO_2_-1 and Fe_3_O_4_@SiO_2_-2 samples were capable of producing higher repulsive forces that compete with the magnetic dipolar attractive forces, thus forming very stable dispersions in water. Hence, their hydrodynamic diameters change only slightly with increasing the concentration of MNPs (Figure S3b). All samples recorded a decrease in their PDI value with increasing the concentration in colloidal solutions. The samples Fe_3_O_4_@SiO_2_-1 and Fe_3_O_4_@SiO_2_-2 exhibit an insignificant decrease in PDI values to 0.22 and 0.17, respectively *(*Table S1*)*. This tiny difference of 0.01 suggests that the sFe_3_O_4_ MNPs from these two samples are stable against aggregation. For the bare Fe_3_O_4_ MNPs and Fe_3_O_4_@SiO_2_-3, the PDI values significantly decrease to 0.23 and 0.26, respectively. This observation together with the important increase in their hydrodynamic diameter indicates a spontaneous aggregation of silica-coated clusters in the case of these two samples with increasing their concentration in colloidal solutions.

### 3.3. Magnetic Characterization of sFe_3_O_4_

**Figure 4:**
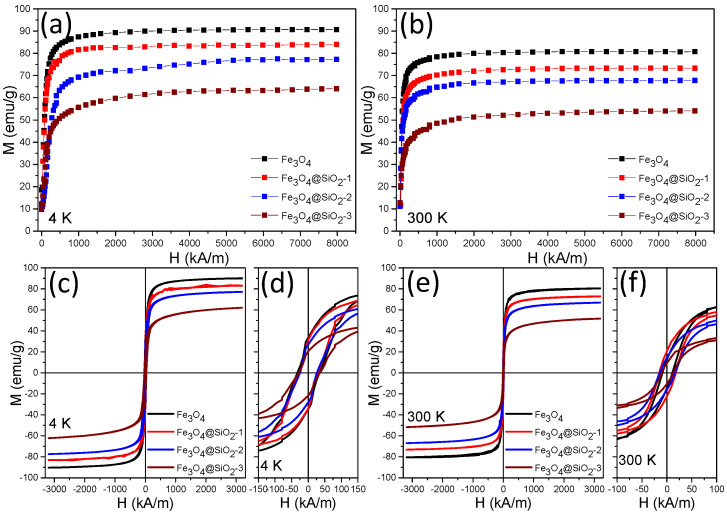
M(H) curves recorded at (**a**) 4 K and (**b**) 300 K, hysteresis loops recorded at (**c**) 4 K and (**e**) 300 K, and low-field regime of hysteresis loops at (**d**) 4 K and (**f**) 300 K for all four types of MNPs.

The magnetic properties of bare Fe_3_O_4_ MNPs and silica-coated MNPs were measured on powders through M-H curves and hysteresis loops at 4 and 300 K (Figure 4). The M-H curves recorded in a magnetic field as high as 10 T (8000 kA/m) reveal the value of the saturation magnetization (M_s_) of the samples. As the magnetization is expressed in electromagnetic units on a per-gram basis, the values of M_s_ thus provide information about the silica thickness coating Fe_3_O_4_ MNPs. The bare Fe_3_O_4_ MNPs exhibited, at 4 K, a M_s_ of 90.6 emu/g, very close to that of the bulk magnetite (92 emu/g), suggesting the high quality in terms of crystallinity of the polyhedral Fe_3_O_4_ MNPs (Figure 4a). For the silica-coated Fe_3_O_4_ MNPs, the M-H curves (Figure 4a) showed a decrease in the M_s_ (Table 1) as expected due to the mass of non-magnetic silica coating. The M_s_ decreased proportionally with the amount of SiO_2_ surrounding the magnetic core: 83.5 emu/g for Fe_3_O_4_@SiO_2_-1, 77.1 emu/g for Fe_3_O_4_@SiO_2_-2, and 63.5 emu/g for Fe_3_O_4_@SiO_2_-3. The differences in the M_s_ of the sFe_3_O_4_ MNPs in respect to bare Fe_3_O_4_ MNPs indicate the following SiO_2_ content within the silicated samples: 7.8% for Fe_3_O_4_@SiO_2_-1, 14.9% emu/g for Fe_3_O_4_@SiO_2_-2, and 29.9% for Fe_3_O_4_@SiO_2_-3. As is typical for MNPs, the M_s_ decreases considerably, at room temperature, due to the existence of the magnetic dead layers and spin canting effects at the surface of MNPs (Figure 4b). In each case, the M_s_ value is reduced by about 10 emu/g (Table 1), which indicates that this is due to the magnetic core and not to the SiO_2_ shell.

Hysteresis loops obtained at 4 K (Figure 4c,d) show that the coercive field (H_c_) does not vary significantly over the four samples (Table 1). Concerning the bare Fe_3_O_4_ MNPs, which exhibit the highest H_c_ of 32 kA/m, a minute decrease in H_c_ is recorded for sFe_3_O_4_ MNPs: 28 kA/m for Fe_3_O_4_@SiO_2_-1, 26 kA/m for Fe_3_O_4_@SiO_2_-2, and 30 kA/m for Fe_3_O_4_@SiO_2_-3 (Table 1). As shown in our previous paper [53], the polyhedral Fe_3_O_4_ MNPs preserve a soft ferromagnetic character at room temperature, which is also present on sFe_3_O_4_ MNPs (Figure 4e,f). The values of H_c_ decrease at room temperature for all four samples as indicated by the hysteresis loops (Figure 4e,f). The highest drop of H_c_ is recorded for bare Fe_3_O_4_@SiO_2_ among all samples (Table 1). The ferromagnetic bare Fe_3_O_4_ MNPs form clusters, as shown by DLS measurements, whereas the strong dipole coupling interaction between clusters tends to assist magnetization reversal, thereby reducing the H_c_ value [55]. The Fe_3_O_4_@SiO_2_-1 and Fe_3_O_4_@SiO_2_-2 samples exhibit the highest H_c_ values at room temperature of 18 and 16 kA/m, respectively, followed by the Fe_3_O_4_@SiO_2_-3 sample with a slightly lower H_c_ value of 12 kA/m (Table 1). Although the difference in H_c_ between silica-coated samples and bare Fe_3_O_4_ MNPs is not important, it can be related to the silica shell protecting the magnetic clusters. The increase in the H_c_ is a consequence of a decrease in the magnetic dipole coupling interactions between clusters due to the screening of silica shell coating [33].

### 3.4. Magnetic Hyperthermia Properties of sFe_3_O_4_

The magnetically induced heating capabilities of all four samples were measured in water (the heating curves are shown in Section S4). Specific absorption rate (SAR) values were obtained using Box–Lucas fitting [56] of the heating curves, following the procedure detailed in Section S5. The SAR values have been expressed as a function of the amplitude (H) of the applied alternating magnetic field ranging from 5 to 65 kA/m at a fixed frequency of 355 kHz. The iron concentration of MNP aqueous solutions was determined with a thiocyanate assay and used for data normalization (Section S6), expressing the SAR in watts per unit mass of iron (W/g_Fe_). In the SAR determination, the contribution from pure water at each H was measured and subtracted as the background.

**Figure 5:**
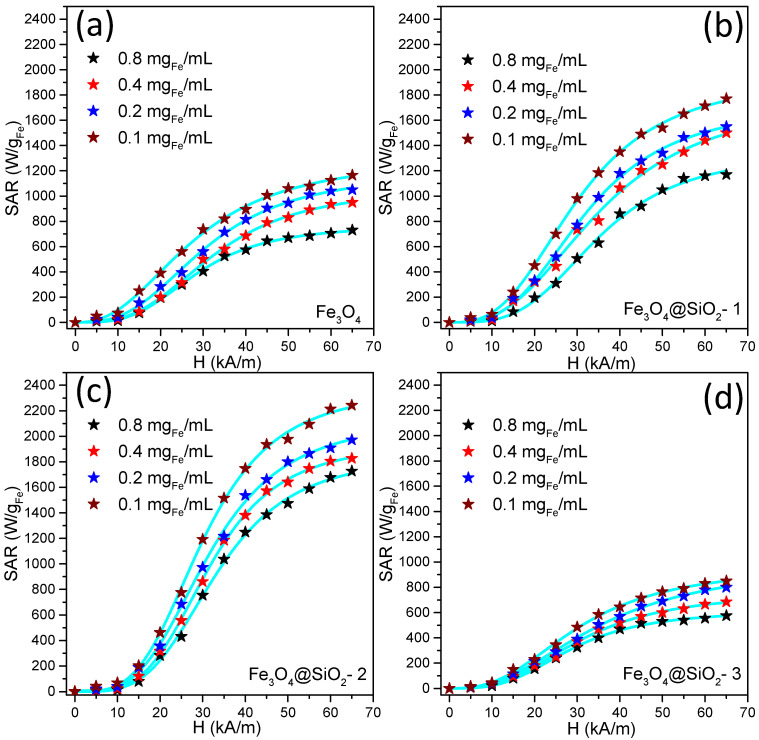
Specific absorption rate (SAR) dependence of H for (**a**) Fe_3_O_4,_ (**b**) Fe_3_O_4_@SiO_2_-1, (**c**) Fe_3_O_4_@SiO_2_-2, and (**d**) Fe_3_O_4_@SiO_2_-3 MNPs dispersed in water at iron concentration ranging from 0.8 to 0.1 mg_Fe_/mL. Blue lines represent the fits with the logistic function.

Figure 5 summarizes the SAR values obtained in water for all four samples presented in this study. Each panel displays the mean SAR values as a function of H for the case of four different iron concentrations, ranging from 0.8 to 0.1 mg_Fe_/mL. It has to be mentioned that in the case of ferri/ferromagnetic MNPs, the main contribution to their hyperthermia capabilities is given by the energy losses quantified by the area of hysteresis loops. Additionally, the Brown relaxation—the reorientation of the MNPs itself in a fluid, resulting in their friction with the fluid—also contributes as a function of the medium viscosity. Hysteresis losses depend on the H and H_c_ of the MNPs. For low values of H, the hysteresis areas are very small and, consequently, the SAR values are insignificant. For H values greater than H_c_, the hysteresis area becomes larger as H increases, resulting in a steep increase in the SAR values up to a saturation value. Further increase in H will lead to a plateau of the SAR values. This typical evolution of SAR values with H (Figure 5) presents a sigmoidal shape that can be fitted (blue curves in Figure 5) phenomenologically with a simple logistic function (Section S7) as shown in our previous papers [24,53,57,58].

The SAR evolution with H for bare Fe_3_O_4_ MNPs reveals a slight dependence on the concentration (c) of MNPs in aqueous solution (Figure 5a). For a c = 0.8 mg_Fe_/mL the SAR values increase sigmoidally, reaching around 700 W/g_Fe_ at the highest H values. With decreasing c by half, the SAR values do not change for H between 5 and 35 kA/m. Starting with H = 40 kA/m, higher SAR values are obtained, with a maximum of 950 W/g_Fe_ at the highest H. The SAR values continue to increase in the entire range of H as the concentration is further decreased to 0.1 mg_Fe_/mL (Figure 5a). In the literature, this behavior is associated with a decrease in the magnetic dipolar interactions between the MNPs as the concentration decreases [59,60]. A closer look at the panels in Figure S4a reveals the occurrence of kinks in the heating curves at each H, which are less pronounced as the c decreases. The kinks reduce the initial slopes of the heating curves leading to a decrease in SAR values. The kinks denote a spontaneous aggregation of MNPs clusters during the hyperthermia measurements.

For the case of Fe_3_O_4_@SiO_2_-1 MNPs, the SAR values’ dependence on the concentration followed the same trend observed for bare Fe_3_O_4_ MNPs (Figure 5b), which means as the c decreases the SAR values increase mainly starting at H = 15 kA/m. The differences became more pronounced as the H increased. The kinks are observed in the heating curves at 0.8 mg_Fe_/mL, disappearing at lower concentrations (Figure S4b). This implies the absence of aggregation effect under AMF in the case of more diluted samples, resulting in the highest SAR values at the smallest concentration. The decrease in the heating curves slopes at 0.8 mg_Fe_/mL, upon the formation of kinks, is less evident for Fe_3_O_4_@SiO_2_-1 MNPs with respect to bare Fe_3_O_4_ MNPs, which suggests that the thin silica layer protects the clusters from making physical contact to form large aggregates responsible for decreasing SAR values. As compared to bare Fe_3_O_4_ MNPs, the SAR values of Fe_3_O_4_@SiO_2_-1 MNPs are higher for each concentration, which can be explained by the better colloidal stability conferred by the thin silica layer. The difference in SAR values between the first two samples occurs starting with 30 kA/m, at each concentration. In the region of high H (50–60 kA/m), the difference increases from 450 to 600 W/g_Fe_ as the concentration decreases from 0.4 to 0.1 mg_Fe_/mL.

Among all the samples studied, the Fe_3_O_4_@SiO_2_-2 MNPs display the highest SAR values over the H range between 20 and 65 kA/m (Figure 5c). Concerning the bare Fe_3_O_4_ MNPs, the difference between SAR values progressively increases with H (compare Figure 5a,c), attaining a difference around 1000 W/g_Fe_ at the highest H (65 kA/m) for all four concentrations. Similar to the previous two samples, the SAR values of Fe_3_O_4_@SiO_2_-2 MNPs strongly depend on the concentration: the maximum value of SAR increases from 1700 W/g_Fe_ at 0.8 mg_Fe_/mL up to 2200 W/g_Fe_ for 0.1 mg_Fe_/mL. The Fe_3_O_4_@SiO_2_-2 MNPs exhibit a well-defined silica layer around them and the highest zeta potential (Figure 3c) as compared to Fe_3_O_4_@SiO_2_-1 MNPs, which confer them excellent colloidal stability. This is evidenced by the absence of kinks in the heating curves for the entire concentration range (Figure S4c). Moreover, from Table S1, it can be observed that the exponent n—which indicates how steep is the dependence of SAR on H—presents the highest values among all samples for each concentration. This suggests that the heating behavior of these types of MNP is closer to an ideal Stoner Wohlfarth model [61]. In other words, the silica layer protects the clusters of MNPs from aggregation and reduces considerably the magnetic dipolar interactions among them, mainly at lower concentrations where the mean inter-clusters distance is high.

A thicker layer of silica around Fe_3_O_4_ MNPs, as in the case of Fe_3_O_4_@SiO_2_-3 MNPs, offers a completely different picture of heating capabilities. As can be seen in Figure 5d, the SAR values decrease with respect to those recorded for bare Fe_3_O_4_ MNPs for each concentration. The reduction in SAR values is explained by the sedimentation of Fe_3_O_4_@SiO_2_-3 MNPs at the bottom of the vial, as observed during measurements. The small value of the zeta potential (Figure 3c) leads to the aggregation of Fe_3_O_4_@SiO_2_-3 MNPs, thus reducing their Brownian mobility and increasing their dipolar interactions.

We also performed SAR measurements with the MNPs dispersed in a solid matrix. Polyethylene glycol 8000 (PEG *K) with a melting point above 50 °C was used as a solvent. PEG 8K was heated to 90 °C and the MNPs were dispersed in the solved under sonication. The sample was allowed to cool at room temperature and afterward was submitted to the various AMF. As can be seen from Figure S6 panel a, the best SAR performances were recorded again for the sample Fe_3_O_4_@SiO_2_-2 with a maximum SAR around 1200 W/g_Fe_. In addition, for this sample, we calculated the maximum drop in the SAR (from 1800 to 1200 W/g). This result indicates also that this sample has the largest mobility among all samples. Interestingly, when the SAR values were recorded as a function of the concentration (Figure S6 panel b) it appeared that the heating performances of these NPs do not depend on concentration. This result can be explained by the fact that the NPs are well dispersed in the matrix and after solidification, the solid matrix hinders the clusterization of the MNPs under the influence of the AMF field.

Among the four samples, the Fe_3_O_4_@SiO_2_-2 MNPs, both dispersed in an aqueous solution or in a solid matrix, exhibited the best MH performance. In this regard, to verify their potential in MH applications, this class of MNPs was further tested in vitro on cancer and normal cell lines.

### 3.5. Cytocompatibility of Fe_3_O_4_@SiO_2_-2 MNPs and Intracellular Modifications

The evaluation of in vitro cytocompatibility of Fe_3_O_4_@SiO_2_-2 MNPs represents an essential step in their validation for MH therapy applications. Alamar Blue (AB) and Neutral Red (NR) assays were employed for cytocompatibility evaluation on two types of cancer cell lines (human pulmonary cancer cells—A549 and melanoma cancer cells—A375) and one normal cell line (human foreskin fibroblasts—BJ). Because the nanomaterials are prone to induce interference with the biochemical assays used to evaluate viability, leading to erroneous results [62], in a first step, the optical and biochemical interferences of the Fe_3_O_4_@SiO_2_-2 MNPs with both assays were evaluated (Section S9). Similar to bare Fe_3_O_4_ MNPs [53], the emitted fluorescence gradually decreases as the concentration of Fe_3_O_4_@SiO_2_-2 MNPs increases, for both assays (Figure S7). The presence of Fe_3_O_4_@SiO_2_-2 MNPs quenches the emitted fluorescence, which can be optically avoided by the measurement of the supernatant fluorescence after removing the MNPs by centrifugation (Figure S7). On the contrary, no interference by reduction/oxidation or adsorption of the dyes was recorded for both assays (Figure S8).

**Figure 6:**
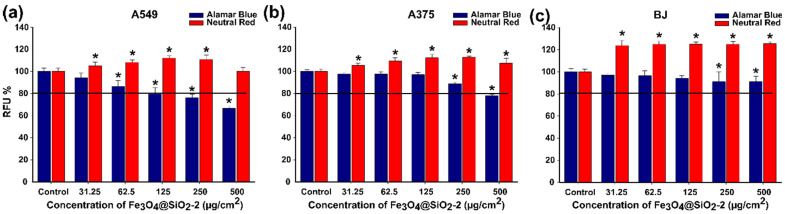
Cytocompatibility of Fe_3_O_4_@SiO_2_-2 MNPs on (**a**) A549, (**b**) A365, and (**c**) BJ evaluated after 24 h exposure. Cellular viability was measured using two complementary assays, namely Alamar Blue and Neutral Red. The values are expressed as mean ± SD of six biological replicates. Data are expressed as relative values to the negative control. Asterisks (*) indicate significant differences compared to the negative control (ANOVA + Dunn’s; *p* < 0.05). The horizontal straight line indicates the cell viability threshold of 80% to consider MNPs as biomedical safe.

The Fe_3_O_4_@SiO_2_-2 displayed a low cytotoxicity profile towards the normal and cancerous cell phenotypes. Different viabilities were recorded, depending on the assay used, with the AB assay displaying a higher sensibility than the NR assay. In the case of the AB assay, a statistical decrease in viability was recorded starting from an exposure dose of 62.5 µg/cm^2^ for A549 cells and 250 µg/cm^2^ for A375 and BJ cells (Figure 6). However, considering the viability threshold of 80% for nanomaterials to be safe for biomedical applications [63], the Fe_3_O_4_@SiO_2_-2 MNPs show a minor toxic behavior at the dose of 250 µg/cm^2^ for A549 cells and 500 µg/cm^2^ for A375 cells, and were nontoxic for BJ cells over the tested dose range (Figure 6). The results indicate that normal cells are more resilient to the toxicity elicited by Fe_3_O_4_@SiO_2_-2, in agreement with our previously published results, where the toxicity of polyhedral iron oxide magnetic nanoparticles (IOMNPs) was slightly more pronounced on the A549 cell line than on normal human gingival fibroblast (HGF) cells [53]. In comparison with normal cells, cancerous cells show an increased ability to uptake MNPs, resulting in a higher cytotoxic susceptibility. The silica shell surrounding the MNPs slightly improves the biocompatibility as compared to citric acid-coated Fe_3_O_4_ MNPs. The viability of A549 cells decreases below 80% for a dose of 125 µg/cm^2^ of Fe_3_O_4_@SiO_2_-2 MNPs (Figure 6a), whereas in the case of citric acid-coated Fe_3_O_4_ this happens at a dose of 600 µg/mL (equivalent to 120 µg/cm^2^) [53]. Conversely, in the case of NR assay, an overall increase in fluorescence was registered (Figure 6). As the NR assay is based on the ATP-dependent incorporation of the NR dye in the lysosomal compartment, we hypothesize that exposure to Fe_3_O_4_@SiO_2_-2 increases the lysosomal compartment and thus the accumulation of the dye. These differences between formazan-related assays (AB, MTT, MTS) and the NR assay were previously reported in the literature by our group and others [53,62].

In the following, the internalization of Fe_3_O_4_@SiO_2_-2 MNPs in different doses (15.62, 31.25, and 62.5 µg/cm^2^) by all three types of cells is presented, aiming at revealing the internalization mechanism and possible intracellular modifications. Qualitative and some quantitative ultrastructural aspects are reported here for each experimental group of cells (for a detailed statistical analysis see Table 2).

First, untreated control cells from all three types of cells were visualized by both SEM and TEM. As shown and described in Section S10, the cells were adherent, presenting the characteristic shapes and internal structures (Figure S9).

**Figure 7:**
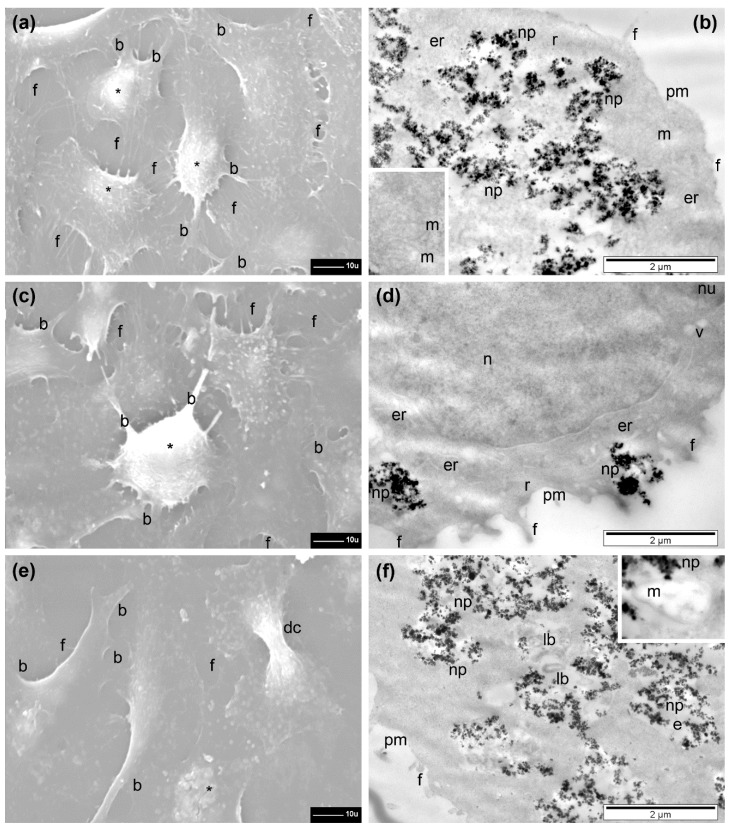
SEM (**left**) and TEM (**right**) images of A549 cells containing Fe_3_O_4_@SiO_2_-2 MNPs after 24 h incubation time in a dose of (**a**,**b**) 15.62 µg/cm^2^, (**c**,**d**) 31.25 µg/cm^2^ and (**e**,**f**) 62.5 µg/cm^2^. The significance of letters is: f = filopodia, b = branch, np = nanoparticles, er = endoplasmic reticulum, r = ribosome, m = mitochondria, pm = plasma membrane, n = nucleus, nu = nucleolus, v = vacuole, dc = divided cell and lb = lamellar bodies. The insets show (**b**, left) mitochondria and (**f**, right) mitochondria and free nanoparticles in cytoplasm. The asterisks indicate retracted cells. 10 u = 10 μm.

The A549 cells generally preserved their adherence and the flat shape with a normal aspect and a with many filopodia still present upon incubation with Fe_3_O_4_@SiO_2_-2 MNPs at doses of 15.62, 31.25, and 62.5 µg/cm^2^ (Figure 7a,c,e). However, a tendency of retraction was obvious starting with a lower dose, resulting in reduced sizes of cells and prominence of their central region (Figure 7a), visualization of thicker branches (with statistical relevance when compared to control cells), and a progressive (significant) reduction in the number of filopodia, with the dose (also significant) when compared to control. The number of retracted cells increased with the exposed dose of Fe_3_O_4_@SiO_2_-2 MNPs. Their shapes were almost spherical, with a low number of thin branches and almost no filopodia; those remaining filopodia were very short and thickened (Figure 7c,e). In the regions where the cells were fewer, the filopodia were even longer (Figure 7a). Cells during division were also observed (Figure 7c).

TEM examination revealed the presence of high amounts of Fe_3_O_4_@SiO_2_-2 MNPs inside cells even starting with the dose of 15.62 µg/cm^2^ (Figure 7b). A variation of the internalized amount of Fe3O4@SiO2-2 MNPs with increasing the dose was not evident in TEM investigations (Figure 7b,d,f). The Fe_3_O_4_@SiO_2_-2 MNPs were internalized in large endosomes (electron-lucent polymorphous vesicles surrounded by a single membrane) and released within the unstructured fraction of cytoplasm (Figure 7b and Figure S10). However, the number of free Fe_3_O_4_@SiO_2_-2 MNPs dispersed in cytoplasm increased with increasing dose (Figure 7d,f). At a dose of 62.5 µg/cm^2^, a few Fe_3_O_4_@SiO_2_-2 MNPs were also found inside lysosomes (homogeneous or heterogeneous electron-dense organelles, surrounded by a single membrane, usually round, not shown—see Figure S10). The nucleus was not affected at all and the endoplasmic reticulum (which appeared as shorter or longer membranous channels, with or without ribosomes attached) displayed a slight enlargement at a dose of 31.25 µg/cm^2^ (Figure 7d), and was difficult to detect at a higher dose (Figure 7f). The mitochondria (with double membrane—the inner one with internal foldings called cristae) preserved a round-oval shape and their cristae had a normal ultrastructural aspect (Figure 7b), becoming swollen, with electron-lucent matrix and altered cristae at the dose of 62.5 µg/cm^2^ (inset Figure 7f). At this dose the cells were more affected by MNPs’ interaction, displaying rarefied cytoplasm, many large vacuoles, and, in rare cases, even disrupted plasma membrane (not shown), resulting in cell death as indicated by AB and NR assay (Figure 6a).

Most of the A375 cells displayed a retraction process due to the interaction with the MNPs, appearing to be smaller in size and more prominent, and were still adherent on the glass substrate (Figure S11a,c,e). Their branches were significantly fewer as compared to the control. Similar to the case of A549 cells, the percent of retracted A375 cells increased by increasing the exposure dose. As compared to those of the untreated control cells (Figure S9c), the filopodia of retracted cells were shorter and in reduced number (Figure S11a,c,e), along with a general progressive (significant) decrease in their mean number. Rare structures resembling lamellipodia were noticed at the periphery of some cells, also bearing filopodia, but they were likely only thinned regions of the cells presenting this unusual aspect during the cellular retraction (Figure S11a). Some of the filopodia had normal length establishing contacts between the cells separated during retraction (Figure S11e).

A high quantity of internalized Fe_3_O_4_@SiO_2_-2 MNPs independent of the exposed dose was observed (Figure S11b,d,f). At a dose of 15.62 µg/cm^2^, the Fe_3_O_4_@SiO_2_-2 MNPs were seen inside endosomes, and dispersed, in direct contact with the aqueous medium of cytoplasm (Figures S10 and S11b). By increasing the dose, clusters of Fe_3_O_4_@SiO_2_-2 MNPs of various sizes dispersed in cytoplasm and rare endosomes containing Fe_3_O_4_@SiO_2_-2 MNPs were identified (Figure S11c,f). Neither relevant ultrastructural changes of the nucleus nor of the endoplasmic reticulum were found for the three doses. However, starting with a dose of 15.62 µg/cm^2^, a mitochondrial reaction to the presence of Fe_3_O_4_@SiO_2_-2 MNPs was identified, which consisted of swelling of some mitochondria, and also showed electron-lucent matrix and cristae alteration (Figure S11b). Mitochondria had an electron-lucent matrix and showed a tendency of cristae disorganization at the dose of 31.25 µg/cm^2^ (Figure S11d), whereas at the higher dose the mitochondria were polymorphous, their matrix was rarefied, but cristae were still visible (Figure S11f). Two other important ultrastructural reactions were noted at a dose of 31.25 µg/cm^2^: the presence of vacuoles in high numbers (and of various sizes), and the presence of lamellar bodies in a relatively high number (Figure S11d,f). It seems that the A375 cells are slightly more sensitive to the interaction with Fe3O4@SiO2-2 MNPs than A579 cells.

**Figure 8:**
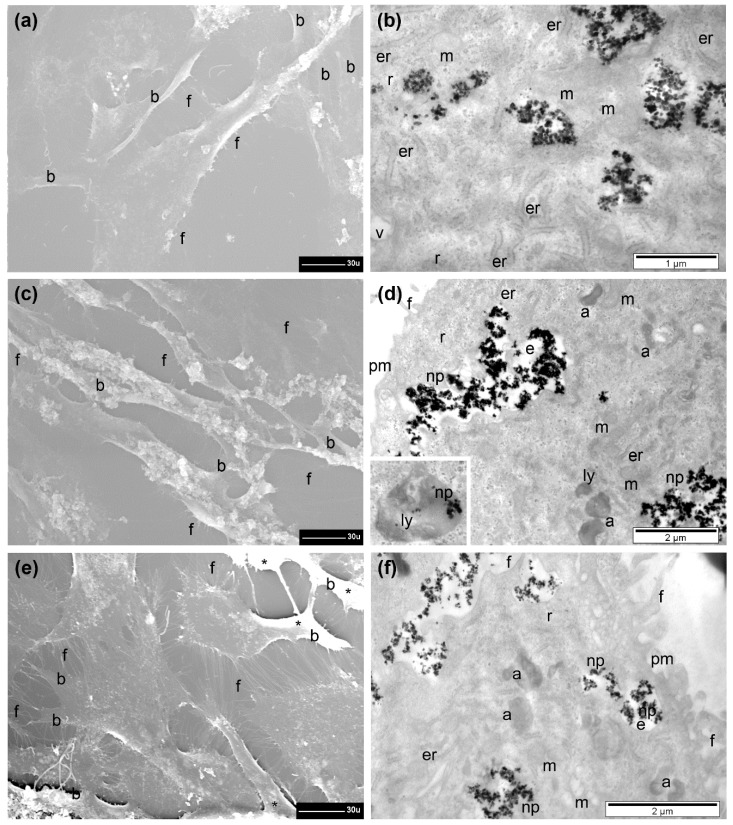
SEM (**left**) and TEM (**right**) images of BJ cells containing Fe_3_O_4_@SiO_2_-2 MNPs after 24 h incubation time in a dose of (**a**,**b**) 15.62 µg/cm^2^, (**c**,**d**) 31.25 µg/cm^2^ and (**e**,**f**) 62.5 µg/cm^2^. The significance of letters is: f = filopodia, b = branch, np = nanoparticles, er = endoplasmic reticulum, r = ribosome, m = mitochondria, v = vacuole, pm = plasma membrane, e = endosome, ly = lysosome and a = autophagosome. The inset show (**d**, left) a lysosome with nanoparticles. The asterisks indicate retracted cells. 30 u = 30 μm.

The BJ cells were adherent and generally preserved their size, and filopodia were present in high number at their surface. Between two and five branches of various sizes were still visible when incubated with the small dose of 15.62 µg/cm^2^ of Fe_3_O_4_@SiO_2_-2MNPs (Figure 8a), comparable to the control group. A retraction tendency of cells (more than 30%) was observed when increasing the dose to 31.25 µg/cm^2^, displaying an elongated shape with significantly fewer visible branches and reduced number of filopodia (significant when compared to untreated control cells and to the previous dose) (Figure 8c). At the highest dose tested, most of the BJ cells showed a normal shape and aspect; however, many cells were visibly retracted. Normally developed filopodia were present in high numbers at the surface of the normal-looking cells, and in the less retracted regions of the other cells (Figure 8e).

Similar to both types of malignant cells, the Fe_3_O_4_@SiO_2_-2 MNPs were internalized by the normal cells in high quantities: packed in large endosomes, dispersed freely in cytoplasm, or captured inside lysosomes (Figure 8b,d,f). No ultrastructural reactions were observed at the nuclear level (not shown), and in cytoplasm, a reduced number of autophagosomes (polymorphous and heterogeneous electron-dense vesicles surrounded by two or several membranes, belonging to the lysosomal system) was found, as compared to the untreated control cells (Figure 8b). By increasing the dose from 15.62 to 62.5 µg/cm^2^, autophagosomes were present in high numbers and no notable ultrastructural changes were detected (Figure 8d). The only notable ultrastructural change found, at the higher dose, was that some mitochondria appeared to be swollen and with disrupted cristae (Figure 8f). It is quite evident that normal cells are less sensitive to the interaction with Fe_3_O_4_@SiO_2_-2 MNPs than malignant cells, in agreement with the AB assay (Figure 6c). The overall increase in fluorescence, displayed by the NR assay, for the malignant cells (Figure 6a–c), can be explained by the increased number of autophagosomes, which enable the accumulation of a large amount of NR dye in lysosomes.

The cellular effects of the Fe_3_O_4_@SiO_2_-2 MNPs’ internalization in the three types of cells tested in our study were consistent with our results proving their cytocompatibility. We recorded moderate surface and internal ultrastructural alterations, both in the two lines of malignant cells (A549 and A375) and in the normal cells (BJ), mostly compatible with life. Even the percentage of the retracted cells was similar in the three lines (about 40%). It is worth noting here that we considered as retracted the cells ranging from those still adherent (without or with branches) and with their central zone more prominent (or more prominent branches), up to completely spherical cells, and all cells still viable despite their changes. Of course, our EM investigation revealed a low number of cells that responded more severely to the presence of the Fe_3_O_4_@SiO_2_-2 MNPs. It is very likely that those cells, along with other dead/fragmented cells (probably lost during repeated centrifugations performed) covered a percentage of 5–15% of the cells, as also resulted from the cytocompatibility tests and literature data.

### 3.6. Quantitative Assessment of Fe_3_O_4_@SiO_2_-2 MNPs Internalization

**Figure 9:**
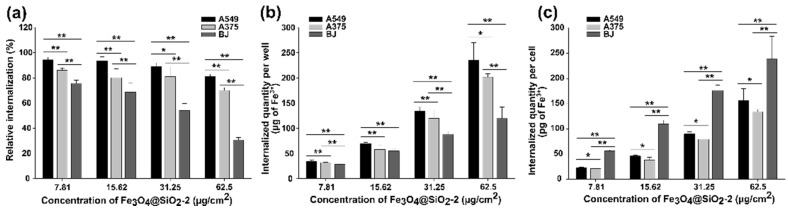
(**a**) The relative internalization (ratio between the internalized amount and the exposed amount) in A549, A375, and BJ; (**b**) the total amount of Fe^+3^ internalized per well; (**c**) the amount of Fe^+3^ internalized per cell evaluated after 24 h exposure to different concentrations of Fe_3_O_4_@SiO_2_-2. The values are expressed as mean ± SD of at least three biological replicas. * *p* < 0.05 ** *p* < 0.001.

Different internalizations were observed between the cell lines, with the normal cell phenotype displaying the lowest internalization, independent of the dose used (Figure 9). As a general trend, for all three types of cells, the relative internalization increased with decreasing doses (Figure 9). For the lowest dose (7.81 µg/cm^2^), the relative internalization for A549 cells was 95%—meaning that almost all amount of Fe_3_O_4_ MNPs was internalized —slightly decreasing to 82% for the highest dose (62.5 µg/cm^2^). In the case of A375 cells, the relative internalization ranged between 85% and 70% by increasing the dose from 7.81 to 62.5 µg/cm^2^. The normal BJ cells displayed the highest drop (40%) in the relative internalization, from 75% to 30% as the dose is increased from 7.81 to 62.5 µg/cm^2^. The differences in the relative internalization are reflected in the total amount of internalized Fe^3+^, which is the highest for the A549 cells, followed by A375 and BJ cells (Figure 9b). For the first two doses (7.81 and 15.62 µg/cm^2^), the differences in the amount of Fe^3+^ internalized by the three types of cells are negligible (Figure 9b and Table S3). The differences are more pronounced as the dose increases (Figure 9b and Table S3). Statistical analysis (two-way ANOVA + Holm–Sidak) revealed that both the dose variable (*p* < 0.001) and the cellular type variable (*p* < 0.001) had a highly significant impact on the Fe_3_O_4_@SiO_2_-2 cellular internalization. It is worth mentioning that, compared with citric acid-coated Fe_3_O_4_ MNPs, which exhibited a relative internalization between 37% and 21% for a dose ranging from 10 to 100 µg/cm^2^ for A549 cells [53], the Fe_3_O_4_@SiO_2_-2 MNPs display from 2.5 to 3.2 times higher relative internalization over the same dose range. This can be because the silica layer prevents the formation of micrometer-sized MNPs aggregates in cell culture media, thus leading to a homogeneous dispersion of sFe_3_O_4_ clusters over the entire well surface paved with cancer cells, which facilitate the internalization of a higher amount of MNPs inside cells.

A common way of expressing the cellular internalization of Fe_3_O_4_ MNPs is to normalize the amount of internalized Fe^3+^ on the cell number. As BJ cells have a higher volume and surface than the cancerous cells, they are less numerous in a well plate and hence internalized a higher amount of Fe^3+^ per cell (Figure 9c). Over the dose range the BJ cells internalized between 50 to 240 pg_Fe_/cell, whereas for the cancer cells the values were between 20 and 150 pg_Fe_/cell (Figure 9c). Recently, Reczynska et al. [40] reported that A549 cells treated with SPIONs coated with a non-porous and mesoporous silica layer at a dose of 10 µg/mL (equivalent to 6.25 µg/cm^2^) internalized approximately 20–25 pg_Fe_/cell. Almost identical values (21, 22 pg_Fe_/cell) were obtained in our study, at the lowest dose of 7.81 µg/cm^2^ on both types of cancer cells. Similar results were also reported for zinc ferrite nanoparticles coated with silica, the Fe^3+^/cell increasing from approximately 5 to 60 pg_Fe_/cell during a 12 h incubation with a dose of 200 µg/mL (equivalent to 125 µg/cm^2^) [38]. Overall, the results obtained suggest a passive transport process, as the amount of internalized Fe_3_O_4_ MNPs was almost linearly dependent on the exposure dose used, and are congruent with the results reported by Matsuda et al. [64], where the internalization of MNPs (≈10 nm) in three histological types of human mesothelioma cells increased linearly with the dose used.

### 3.7. In Vitro Magnetic Hyperthermia

**Figure 10:**
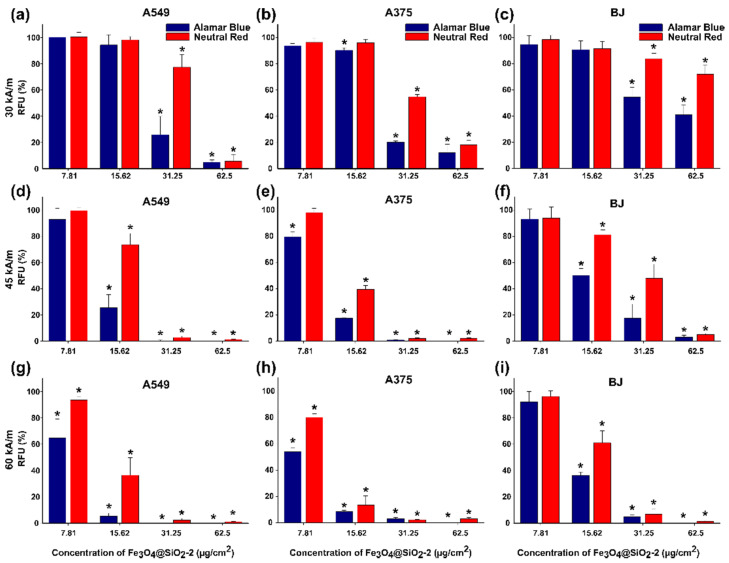
Cell viability (A549-(**a**,**d**,**g**), A375-(**b**,**e**,**h**), BJ-(**c**,**f**,**i**)) after exposure to MNPs and AMF of 30, 45, and 60 kA/m amplitudes. Data are expressed as relative values to the negative control, i.e., cells not exposed to AMF. Asterisks (*) indicate significant differences compared to the negative control (ANOVA + Dunn’s; *p* < 0.05). The values are expressed as mean ± SD of at least three biological replicates.

The in vitro MH on the three types of cells without MNPs did not induce cell death as the temperature rise did not exceed 0.5–0.8 °C for H ranging from 30 to 60 kA/m at 355 kHz. On the contrary, all samples consisting of cells containing internalized MNPs exhibited a relevant increase in the temperature in the first 5 min followed by the formation of a plateau in the heating curves when the heat released by the internalized MNPs equalized the dissipated heat in the environment (Figure S12). The plateau defines a saturation temperature that strongly depends on the amount of internalized MNPs and on H (Figure S12). A correlation between saturation temperature (T_s_) and the viability of cells can be established. For the lowest two doses (7.81 and 12.52 μg/cm^2^), at H of 30 kA/m, no cell death was observed for all three types of cells as evaluated with both toxicity assays (Figure 10a–c). This is explained by the small T_s_ values between 40 and 41.4 °C obtained during MH treatment (Figure S13). At the higher doses of 31.25 and 62.5 μg/cm^2^, the viability of both types of cancer cells, based mainly on the AB assays, was drastically reduced, ranging between 5 and 25%. The internalized MNPs were able to reach T_s_ from 43.5 to 46.4 °C (Figure S13) which suffices the temperature requirements of cancer thermal therapies. For almost the same T_s_ interval, the BJ cells showed a reduction in their viability by only 50–60% (AB assays in Figure 10c), proving that they are less sensitive to magnetic heating as compared to cancer cells.

Increasing the H at 45 kA/m resulted in increased T_s_ values over the entire dose range for all types of cells (Figure 10d–f). Nonetheless, for the lowest dose of 7.81 μg/cm^2^, the cancerous and normal cells were less affected by the MH treatment as T_s_ was around 42 °C (Figure S13). A considerable loss of viability to approximately 20% was recorded starting with a dose of 15.62 μg/cm^2^, mainly for cancerous cells (Figure 10d,e), which is in agreement with the T_s_ value of 44.6/44.7 °C reached during MH treatment (Figure S13). For the same dose, the normal cells exhibited a 50% reduction in their viability (Figure 10f). For the next two doses, the T_s_ increased to values between 48 and 50 °C, leading to cellular death of cancer cells as evidenced by both assays (Figure 10d,e). The normal cells were also profoundly affected by the MH treatment, exhibiting viabilities below 20% (Figure 10f).

The internalized MNPs at the lowest dose (7.81 μg/cm^2^) in both types of cancer cells were able to sustain an increase in T_s_ up to 44 °C, when exposed to an H of 60 kA/m, which resulted in a decrease in viability around 40%, as indicated by the AB assay (Figure 10g,h and Figure S11). A similar quantity of MNP internalized BJ cells did not substantially reduce the viability as the T_s_ increased to 42.2 °C (Figure 10i and Figure S13). The viability dropped to near zero for the next three doses in the case of cancer cells (Figure 10g,h). The BJ cells were less sensitive at a dose of 15.62 μg/cm^2^, as compared to cancer cells, however, their viability was close to zero for the higher two doses (Figure 10i).

**Figure 11:**
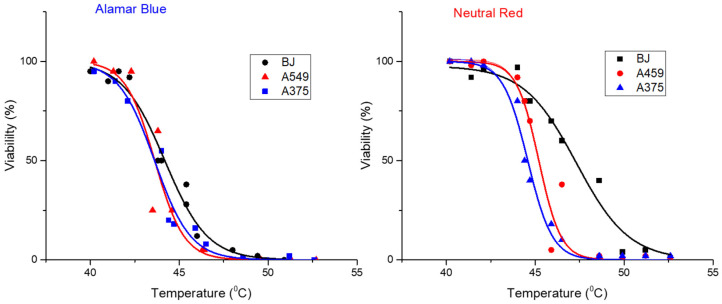
Viability using the Neutral Red (**left** panel) and Alamar Blue (**right** panel) assays as a function of saturation temperatures of cell cultures exposed to Fe_3_O_4_@SiO_2_-2 MNPs and for 30 min to AMF (30, 45, and 60 kA/m, 355 kHz) for the three different cell lines (BJ—black squares, A459—red circles, A375—blue triangles).

For all three cell lines the viability data obtained using both assays could be fitted with a sigmoidal function:

(1) $$C\left(T\right)=\frac{A}{1+e^{\frac{T-T_{0}}{\mathrm{dT}}}}$$

where A represents the viability of control cells (95–100%) and dT quantifies the temperature width for a given decrease in cell viability. T_0_ represents the temperature at which the viability reaches a value of 50%. This equation was derived [65] from a two-state model of temperature-dependent cell damage as initially proposed by Feng et al. [66]. This simplified form was derived for comparing the magnetic hyperthermia heating with endogenous hyperthermia heating, under the condition of having the same time of exposure to high temperatures [65]. As in our case, in all experiments’ cells were exposed for 30 min to MH; the experimental data were well fitted with the function depicted in Equation (1), as can be seen in Figure 11. The temperature T_0_ represents the saturation temperature at which half of the cells were killed after they were exposed for 30 min to the MH treatment or, in other words, the temperature at which the cells were exposed for 30 min to MH receive 50% lethal dose (LD50%). As one can notice with both assays, the T_0_ values are higher for BJ cells as compared to both cancer cell lines, but the difference is significantly higher in the case of the NR assay (Table S4). For this assay, T_0_ is 47.3 °C for normal BJ cells, and 45.2 °C and 44.6 °C for A459 and A375 cells, respectively. The difference is much smaller for the AB assay (44.2 °C for normal and 43.6 °C for both cancer cell lines).

These differences between the LD50% temperature values obtained using the two viability assays might be attributed to their different mechanisms of detection. The NR assay is based on the ATP-dependent lysosomal incorporation of the supravital dye and the measurement of the fluorescence of the incorporated dye. The apparent increase in the viability given by NR assay is most probably the result of an increased lysosomal compartment due to the IOMNPs’ exposure and intra-lysosomal incorporation, as it was also proved by the electron microscopy study. Dissimilarities between viability assays that evaluate different mechanisms of toxicity were previously reported, reiterating the need for multiple viability assays when evaluating the cytocompatibility/cytotoxicity of nanomaterials [67,68]. Similar to the results obtained in this study, a slight decrease in the cellular viability, measured by the WST assay—an assay similar to Alamar Blue—and an increase in the NR-dye uptake upon exposure to IOMNPs were previously reported [62]. Thus, the increased uptake of NR dye may be correlated with the accumulation of IOMNPs in lysosomes. The higher sensitivity of either WST or Alamar Blue viability assay may be related to mitochondrial damage by IOMNPs, which results in a decreased conversion of formazan or resazurin by mitochondrial diaphorase. Moreover, the mitochondrial impairment of nanomaterials is presumed to be related to their redox-active surface that hinders the electron flow and the mitochondrial functionality [69].

Similar to HGF cells [53], the BJ normal cell line is less prone to MH treatment compared to the cancer cell lines. This may be a consequence of a lesser capability to internalize the Fe_3_O_4_@SiO_2_-2 MNPs compared to cancer cells, mainly in the case of the higher doses (31.25 and 62.5 μg/cm^2^). There is no significant difference between the cell viabilities exhibited by the two cancer cell types in the entire range of doses for the three values of H. This is supported by the small differences in saturation temperatures reached during MH, which are mainly due to the almost identical capacities of both types of cancer cells to internalize the Fe_3_O_4_@SiO_2_-2 MNPs (Figure S12). At the lower doses (7.81 and 15.62 μg/cm^2^), for which the total amount of internalized Fe^3+^ by the three types of cells does not vary significantly, the T_s_ recorded for cancer cells are higher than normal ones resulting in appreciable differences between cellular viabilities. This may be explained by the different mobility of internalized silica-coated MNPs on cancer versus normal cells. Inside cancer cells, the silica-coated MNPs can attain higher mobility when subjected to an AMF, as they are less numerous per cell (Figure 9c), leading to an increase in the saturation temperatures, and thus reduced viability.

It is also worth noting that for all cell types and all magnetic field strengths used there is a clear tendency of saturation of the T_s_ (Figure S12). This behavior may be explained by an increase in the concentration of the MNPs taken up by the cells within endosome/lysosome closed structures which can significantly reduce their mobility and thus their heating performance.

### 3.8. Cellular Modification upon MH Exposure of Cells

As in the control, and incubated but unexposed groups of cells, qualitative and some quantitative ultrastructural aspects are presented here, and a detailed statistical analysis is given in Table 2.

#### 3.8.1. A549 Cells

**Figure 12:**
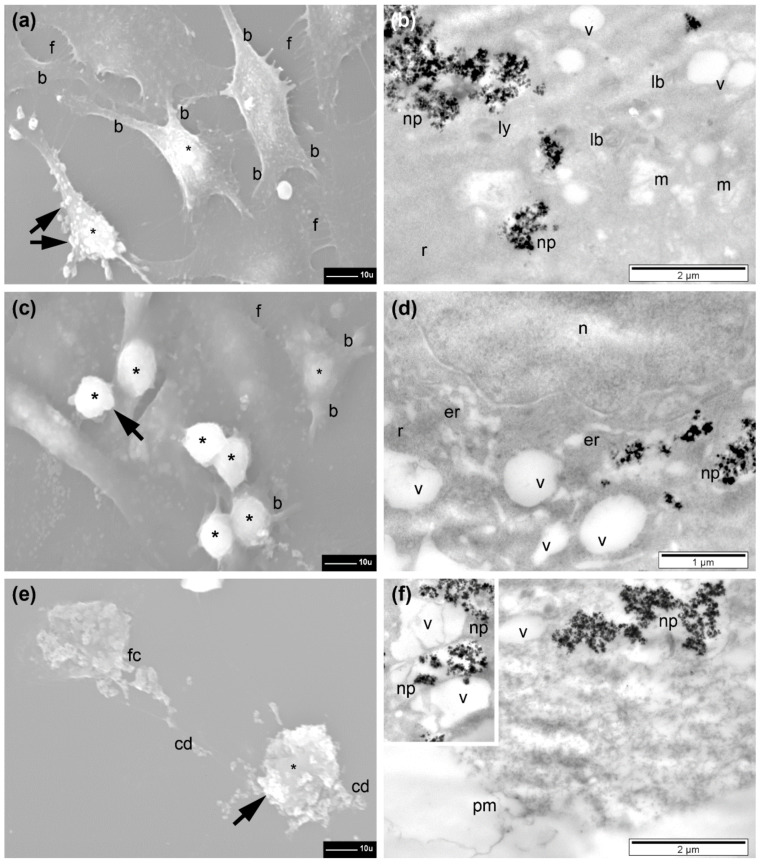
SEM (**left**) and TEM (**right**) images of A549 cells incubated with Fe_3_O_4_@SiO_2_-2 MNPs for 24 h in a dose of (**a**,**b**) 15.62 µg/cm^2^, (**c**,**d**) 31.25 µg/cm^2^, and (**e**,**f**) 62.5 µg/cm^2^ and exposed for 30 min to an AFM of 30kA/m, 355 kHz. The significance of letters is: f = filopodia, b = branch, np = nanoparticles, ly = lysosome, lb = lamellar bodies, v = vacuole, m = mitochondria, r = ribosome, n = nucleus, er = endoplasmic reticulum, pm = plasma membrane, fc = fragmented cells and cd = cellular debris. The inset (**f**, left) show large vacuoles and free nanoparticles in cytosol. The asterisks indicate retracted cells. The black arrows indicate membrane blebs. 10 u = 10 μm.

The exposure of A549 cells, previously incubated with Fe_3_O_4_@SiO_2_-2 MNPs at a dose of 15.62 μg/cm^2^, to an AMF of 30 kA/m for 30 min, resulted in a higher number of retracted cells having significantly more visible branches compared with unexposed incubated cells. Rare contracted and prominent cells showed at their surface many small blebs formed at the level of the plasma membrane. Many other cells were normal, displaying numerous filopodia (Figure 12a); however, the overall mean number of filopodia/cell decreased significantly with regard to both the control group and the group of unexposed cells incubated with the same dose of MNPs (Table 2). Inside the cells, moderate ultrastructural changes were produced, such as mitochondrial alteration (swollen organelles, with electron-lucent cytoplasm and fewer cristae), cytoplasmic vacuolation, and the presence of the lamellar bodies as round or curved vesicles containing several concentric layers of membranes (Figure 12b). The number of normal A549 cells, incubated with the next dose and exposed to AMF of 30 kA/m for 30 min, decreased dramatically. Many other cells (more than 80% of the counted cells) showed accentuated retraction and rounding cells; more than a third of the identified cells were almost spherical, and also very small in diameter (about 10 µm or less). Surprisingly, these round cells had an almost completely smooth surface as seen in SEM, and only rare membrane blebs were observed. The other cells grown on the coverslip were either still flat and completely adherent to the substrate or partially retracted, showing 2–3 thick branches (with a significantly lower mean value than in the cells incubated with the same dose, and the cells incubated with the smaller dose and exposed to the magnetic field).

A similar (and also significant) response was found in this group of cells when we analyzed the number effect of hyperthermia on the number of filopodia. Only the flattest cells still showed filopodia and in small numbers (Figure 12c; Table 2). Cellular reactions were more extensive, consisting of irregular nuclear outline, and enlargement of the perinuclear space and of the endoplasmic reticulum lumen, associated with extensive vacuolation of cytoplasm. In most cells mitochondria were not identified—most likely these swollen organelles having no remaining cristae participated in the generation of the many vacuoles (Figure 12d). Rare normal A549 cells were observed in the case of the 62.5 μg/cm^2^ dose. About a half of all identified cells (53%) were retracted (some still displaying a single visible branch and very few filopodia—Table 2) and rounded, covered with membrane blebs, whereas the other cells were in an advanced stage of fragmentation (Figure 12e). TEM examination confirmed the SEM results: in the smaller pellets remained suitable for analysis, the cells were either fragmented, with rarefied cytoplasm and disrupted plasma membrane on extensive regions (Figure 12f), or, in the case of still-intact cells, they were filled almost completely with numerous and large electron-dense vacuoles (inset of Figure 12f).

#### 3.8.2. A375 Cells

In the case of A375 cells, a stronger response by retraction and plasma membrane blebbing at a dose of 15.62 μg/cm^2^ was found, after MH treatment (Figure S14a,b). The most affected cells were rounded (losing their branches by retraction), and they lost almost completely their adherence to the glass surface. Such highly affected cells were more numerous compared to A549 cells (see Table 2), suggesting a slightly higher sensitivity of these cells to the AMF exposure, which was also indicated by AB assay (Figure 6a,b). Normal-looking cells were also found at SEM examination (Figure S14a). Statistical analysis showed a significantly lower mean number of branches and filopodia in the cells of this group, compared to the corresponding group of unexposed cells but incubated with the MNPs in the same dose (and of course, when compared to the control). The internal reactions of the affected cells were represented by rarefaction of the mitochondrial matrix; swelling of mitochondria and their cristae; cytoplasmic vacuolation; and accentuation of the nuclear polymorphism (Figure S14b). A higher number of cells (over 60%) were rounded and/or almost completely retracted at the dose of 31.25 μg/cm^2^ (Figure S14c). Some cells were in an advanced stage of total fragmentation. A significantly low number of branches (compared to the cells incubated with the same dose), and very few filopodia (compared both to the cells incubated with the same dose and with the cells incubated with the lower dose and exposed to hyperthermia), were observed (average of about 11/cell), regardless of the general aspects of the cells. In the remaining viable cells, which were still adherent and more or less flat, the cytoplasm was filled with vacuoles of various sizes and shapes, some of which resulted from mitochondrial disorganization and swelling, whereas others may result from fragmentation and vesiculation of the endoplasmic reticulum (Figure S14d). Many lamellar bodies were also found. At the dose of 62.5 μg/cm^2^, many of the A375 cells observed on the coverslips were spherical (27.6%), rarely with only one branch and very few filopodia (see Table 2), and the others were retracted and with various degrees of fragmentation. Cellular debris was also noted on the recorded images (Figure S14e). The A375 cells found on the sections at the TEM examination were deeply altered, with a disrupted plasma membrane and extremely rarefied cytoplasm, and sometimes still containing numerous vacuoles of very different sizes (Figure S14f).

#### 3.8.3. BJ Cells

**Figure 13:**
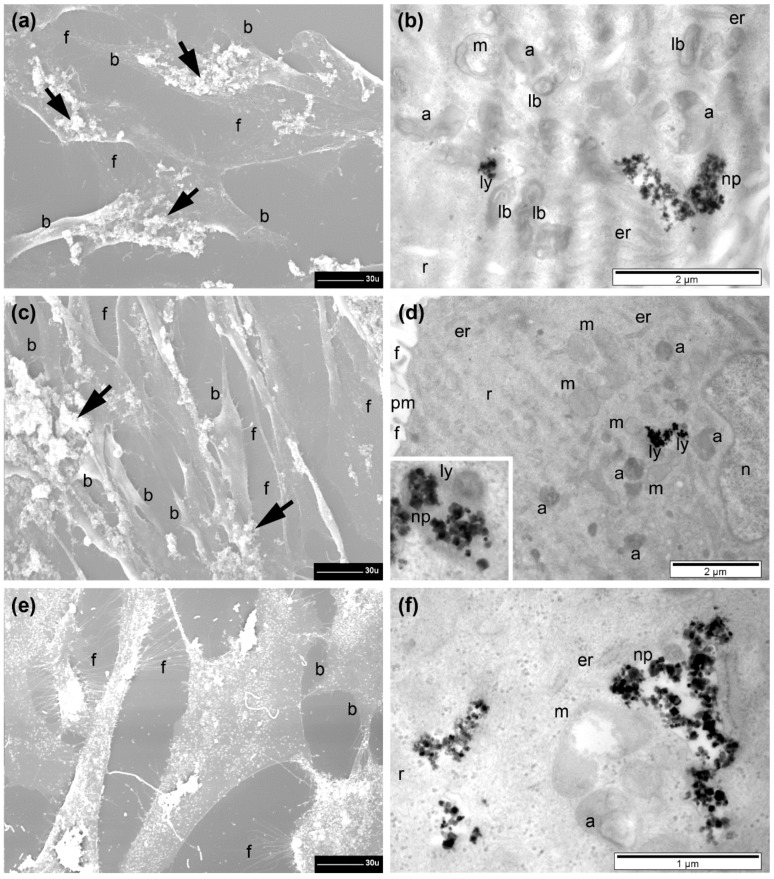
SEM and TEM images of BJ cells incubated with Fe_3_O_4_@SiO_2_-2 MNPs for 24 h in a dose of (**a**,**b**) 15.62 µg/cm^2^, (**c**,**d**) 31.25 µg/cm^2^, and (**e**,**f**) 62.5 µg/cm^2^ and exposed for 30 min to an AMF of 30 kA/m, 355 kHz. The significance of letters is: f = filopodia, b = branch, np = nanoparticles, ly = lysosome, lb = lamellar bodies, m = mitochondria, r = ribosome, er = endoplasmic reticulum, a = autophagosomes and pm = plasma membrane. The inset (**d**, left) show a heterogeneous lysosome loaded with nanoparticles. The black arrows indicate membrane blebs. 30 u = 30 μm.

The BJ cells subjected to incubation with MNPs in the dose of 15.62 μg/cm^2^ and to exposure to AMF also underwent an accentuated retraction and generation of membrane blebs, which were in higher numbers and much smaller compared to those observed in the malignant cells. Normal, but less branched cells (statistically not significant compared to the group of incubated cells with the same dose) were still present (Figure 13a). Over 30% of cells were retracted, and the mean number of filopodia significantly reduced when compared with the corresponding two groups (see Table 2). TEM examination showed a relatively normal ultrastructure, with the presence of numerous autophagosomes and normal profiles of the endoplasmic reticulum. Swollen mitochondria and the presence of rare lamellar bodies were noted (Figure 13b) compared to unexposed BJ cells. At the next dose (31.25 μg/cm^2^), most of the BJ cells were adherent, but appeared as very long and thin cells (a significantly lower average number of branches/cell compared to the three corresponding groups—see Table S2), rather than triangular or flat (Figure 13c). More than half of the cells in this group were retracted. On the surface of their branches, the filopodia were also present but in a significantly lower number only when compared to the control group of BJ cells. Surface membrane blebbing was noted on almost all cells, whereas in some cells this blebbing was extremely advanced, leading to total fragmentation of the cells (Figure 13c). Examination using TEM showed mostly normal ultrastructure of the cells, but mild cellular vacuolation was also found in some cells (Figure 13d), whereas in rare regions only cellular debris was found on the sections (not shown). The BJ cells, loaded with Fe_3_O_4_@SiO_2_-2 MNPs at a dose of 62.5 μg/cm^2^_,_ largely preserved their flat shapes (although some were extremely elongated and thick—significantly fewer than in the previous group, and in the control group, respectively) upon exposure to AMF. Filopodia were present in distinct regions, not covering the whole plasma membrane; the average number of these thin extensions was statistically significant only when compared to the control group. Some of the flat cells showed a slight tendency of retraction (Figure 13e), but overall, we recorded more than 55% of retracted cells. Moreover, 20% (24 out of 120) of the counted cells were fragmented. Regarding internal ultrastructural alterations, only rare mitochondrial deep alterations, and a certain degree of cytoplasmic rarefaction, are worth noting (Figure 13f). Basically, in the case of the BJ cell line, the effect of MH exposure had a much-reduced amplitude as compared with those recorded in the two lines of malignant cells, following the AB and NR assays (Figure 10a–c).

The BJ cells also showed a more variable individual response to the AMF consecutive incubation with the three doses of MNPs, by comparison with the malignant cells. In the BJ group we found cells identical with those in the control group, in addition to others that were highly affected or dead. The reduced average number of branches and/or filopodia was due both to an unspecific reaction of all cells to the experimental conditions and a relatively high number of retracted cells that were more affected. The latter explanation may also apply to the two lines of malignant cells.

### 3.9. Intracellular SAR Values

**Figure 14:**
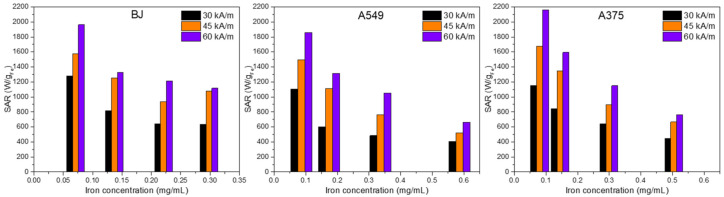
SAR values of the Fe_3_O_4_@SiO_2_-2 MNPs calculated from the saturation temperatures reached during the intracellular MH and the quantity of MNPs inside the cells assessed by the thiocyanate method for BJ (**left** panel), A549 (**middle** panel), and A375 (**right** panel) for three values of the magnetic field intensity (30, 45, and 60 kA/m) at 355 kHz.

The SAR values for the intracellular MH were calculated based on the Box–Lucas equation and the MNPs’ cell uptake data. As can be seen in Figure 14, for all three cell lines there is a clear decrease in the SAR values as the intracellular concentrations of the MNPs increase. This type of dependence was also reported earlier in both normal and cancer cell lines [53].

At the lowest value of the concentration (<0.1 mg/mL), the SAR values are close to those recorded in water. The decrease in the SAR is more pronounced in the case of the cancer cell lines as the quantity of internalized MNPs is almost double that of the BJ normal cancer cell line (Table S3). These results demonstrate that, at very low internalized concentrations, the MNPs preserve mobility comparable to that in water. The SAR decrease with the increase in the MNPs’ concentration may be explained both by the MNPs’ agglomeration in the endosome/lysosome-like structure with a subsequent decrease in their physical mobility (the only heat generation mechanism remaining the magnetization reversal), or the increase in their dipolar interactions due to their proximity.

This type of dependence of SAR on concentration explains the saturation of T_s_ with increasing MNPs’ intracellular concentration presented in Figure S13.

## 4. Conclusions

In this study, ferromagnetic polyhedral Fe_3_O_4_ MNPs were successfully coated with silica shells of different thicknesses by varying the amount of TEOS within the reverse microemulsion technique. The colloidal stability of sFe_3_O_4_ MNPs increased with the silica amount up to a certain critical thickness and then decreased as the silica layer became thicker. The applied silica coating did not affect the intrinsic magnetic properties of the MNPs. The sFe_3_O_4_ MNPs with the highest colloidal stability presented enhanced MH performance in water, and the SAR values increased by almost 1000 W/g_Fe_ compared to bare Fe_3_O_4_ MNPs.

The malignant and normal cells internalized a high quantity of sFe_3_O_4_ MNPs inside large endosomes at a low dose (15.62 μg/cm^2^); the sFe_3_O_4_ MNPs were dispersed in cytoplasm or accumulated inside lysosomes as the dose increased. Cytotoxicity studies using Alamar Blue and Neutral Red assays confirmed the SEM and TEM findings, revealing insignificant toxicity for normal cells over the entire dose range, whereas for A549 and A375 cell lines a drop in cellular viability to 80% was recorded starting with doses of 125 and 250 μg/cm^2^, respectively.

Intracellular magnetic hyperthermia experiments revealed that the malignant cells were more sensitive to MH treatment compared to the normal ones. More than 50% of malignant cells, incubated at a dose of 31.25 μg/cm^2^, underwent cellular death starting with an H of 30 kA/m (355 kHz). Increasing the H to 45 and 60 kA/m enabled the destruction of a large number of malignant cells at lower doses: 15.62 and 7.81 μg/cm^2^, respectively. The affected cells were retracted and rounded, and covered with membrane blebs, displaying rarefied cytoplasm and disrupted plasma membrane on extensive regions, whereas many others were in an advanced stage of fragmentation. In the case of still-intact cells, upon MH treatment, they were filled almost completely with numerous and large vacuoles.

Our data demonstrate that controlled silica coating of ferromagnetic iron oxide nanoparticles significantly increases their hyperthermia performance, cellular uptake, and efficient destruction of cancer cells, making these MNPs excellent candidates for further in vivo studies.

## Figures and Tables

**Table 1:** Magnetic hysteresis parameters at 4 and 300 K for all four types of MNPs.

Sample	M_s_ (emu/g)		H_c_ (kA/m)	
	4 K	300 K	4 K	300 K
Fe_3_O_4_	90.6	80.7	32	10
Fe_3_O_4_-SiO_2_-1	83.5	73.3	28	18
Fe_3_O_4_-SiO_2_-2	77.1	67.5	26	16
Fe_3_O_4_-SiO_2_-3	63.5	53.9	30	12

**Table 2:** Quantitative assessment of several cellular parameters in the experimental groups of A549, A375, and BJ cells. Results are expressed as mean ± standard deviation (ANOVA + Bonferroni correction; statistical significance at *p* < 0.05).

Cell Type	A549			A375			BJ		
Experimental Conditions	Control	Internalization	In Vitro MH Treatment	Control	Internalization	In Vitro MH Treatment	Control	Internalization	In Vitro MH Treatment
**sFe_3_O_4_ dose**	**0**	**15.62 µg/cm^2^**		**0**	**15.62 µg/cm^2^**			**15.62 µg/cm^2^**	
**Cellular branches**	0.76 ± 1.05 (*n* = 103)	2.22 ± 2.05 a (*n* = 100)	2.43 ± 1.82 a (*n* = 56)	4.11 ± 1.41 (*n* = 101)	2.78 ± 1.35 a (*n* = 128)	2.16 ± 1.41 a,b (*n* = 152)	3.93 ± 1.15 (*n* = 86)	3.71 ± 1.16 (*n* = 69)	3.32 ± 1.33 a (*n* = 87)
**Filopodia**	76.69 ± 13.5 (*n* = 83)	77.95 ± 15.7 (*n* = 38)	53.81 ± 22.75 a,b (*n* = 68)	66.49 ± 13.8 (*n* = 85)	35.77 ± 11.1 a (*n* = 86)	24.08 ± 11.2 a,b (*n* = 93)	96.32 ± 23.83 (*n* = 47)	81.43 ± 18.78 (*n* = 61)	51.01 ± 32.76 a,b (*n* = 76)
**Retracted cells** **(% of *n*)**	7.91 (*n* = 215)	17.45 (*n* = 321)	33.33 (*n* = 312)	9.04 (*n* = 166)	30.40 (*n* = 273)	52.74 (*n* = 201)	6.12 (*n* = 147)	10.48 (*n* = 105)	34.74 (*n* = 95)
**sFe_3_O_4_ dose**	**-**	**31.25 µg/cm^2^**		**0**	**31.25 µg/cm^2^**		**0**	**31.25 µg/cm^2^**	
**Cellular branches**	-	2.19 ± 1.62 a (*n* = 73)	1.42 ± 1.44 a,c,e (*n* = 107)	-	2.68 ± 1.23 a (*n* = 122)	1.81 ± 1.42 a,c (*n* = 163)	-	2.79 ± 1.53 a,b (*n* = 106)	2.04 ± 1.18 a,c,e (*n* = 105)
**Filopodia**	-	56.59 ± 19.73 a,b (*n* = 81)	16.45 ± 16.18 a,c,e (*n* = 87)	-	25.72 ± 8.74 a,b (*n* = 75)	11.25 ± 9.99 a,c,e (*n* = 72)	-	58.55 ± 28.95 a,b (*n* = 101)	46.89 ± 33.5 a (*n* = 97)
**Retracted cells** **(% of *n*)**	-	18.01 (*n* = 372)	82.52 (*n* = 452)	-	39.89 (*n* = 188)	63.75 (*n* = 309)	-	31.82 (*n* = 110)	50.26 (*n* = 189)
**sFe_3_O_4_ dose**	**-**	**62.5 µg/cm^2^**		**-**	**62.5 µg/cm^2^**		**-**	**62.5 µg/cm^2^**	
**Cellular branches**	-	2.29 ± 1.44 a (*n* = 151)	0.07 ± 0.26 d,e,f (*n* = 72)	-	2.78 ± 1.35 a (*n* = 145)	0.31 ± 0.81 a,d,e,f (*n* = 65)	-	2.14 ± 1.26 a,b,c (*n* = 140)	1.77 ± 1.77 a,e (*n* = 107)
**Filopodia**	-	33.48 ± 16.58 a,b,c (*n* = 75)	0.63 ± 2.17 a,d,e,f (*n* = 72)	-	24.63 ± 10.1 a,b (*n* = 81)	1.83 ± 4.58 a,d,e,f (*n* = 63)	-	57.1 ± 34.63 a,b (*n* = 83)	44.75 ± 35.61 a (*n* = 101)
**Retracted cells** **(% of *n*)**	-	39.51 (*n* = 243)	100 (*n* = 72)	-	40.29 (*n* = 206)	100 (*n* = 76)	-	42.28 (*n* = 123)	56.67 (*n* = 120)

*n*—counted cells/experimental group; a—significant when compared to control; b—significant when compared to internalization 15.62 µg/cm^2^; c—significant when compared to internalization 31.25 µg/cm^2^; d—significant when compared to internalization 62.5 µg/cm^2^; e—significant when compared to in vitro MH treatment 15.62 µg/cm^2^; f—significant when compared to in vitro MH treatment 31.25 µg/cm^2^.

## Data Availability

All data available are reported in the article and in the supplementary materials.

## Supplementary Materials

The following are available online at https://www.mdpi.com/article/10.3390/pharmaceutics13122026/s1, Figure S1: SEM images of silica-coated Fe_3_O_4_ MNPs, Figure S2: XRD patterns of MNPs, Figure S3: Hydrodynamic diameter of MNPs, Figure S4: Heating curves of silica-coated Fe_3_O_4_ MNPs dispersed in water at different concentrations, Figure S5: Calibration curve for iron concentration determination, Figure S6: SAR dependence on H for MH in PEG 8K, Figure S7: Optical interference of silica-coated Fe_3_O_4_ MNPs with Alamar Blue and Neutral Red assay, Figure S8: Biochemical interferences of silica-coated Fe_3_O_4_ MNPs with Alamar Blue and Neutral Red assays, Figure S9: SEM and TEM images of A549, A375, and BJ cells, Figure S10: Relevant TEM images of BJ and A375 cells incubated with silica-coated Fe_3_O_4_ MNPs, Figure S11: SEM and TEM images of A375 cells incubated with silica-coated Fe_3_O_4_ MNPs, Figure S12: Heating curves of cells incubated with silica-coated Fe_3_O_4_ MNPs, Figure S13: Saturation temperatures, Figure S14: SEM and TEM examination of A375 cells incubated with silica-coated Fe_3_O_4_ MNPs and exposed for 30 min to an AMF of 30 kA/m, 355 kHz. Table S1: PDI indexes as obtained from DLS data, Table S2: Fitting parameters of SAR evolution with H, Table S3: Amount of Fe^3+^ internalized in cells relevant for in vitro cytotoxicity assays and Fe^3+^ concentration of samples used in in vitro magnetic hyperthermia, Table S4: Fitting parameters of cytotoxicity data upon intracellular MH based on Equation (1).
