# Supplementary material for: Silica Coating of Ferromagnetic Iron Oxide Magnetic Nanoparticles Significantly Enhances Their Hyperthermia Performances for Efficiently Inducing Cancer Cells Death In Vitro
Source: Pharmaceutics. 2021 Nov 27;13(12):2026. doi: 10.3390/pharmaceutics13122026 (PMC8706665; doi:10.3390/pharmaceutics13122026)
Supplement: Supplementary file 1 [file pharmaceutics-13-02026-s001.zip › pharmaceutics-1460210-supplementary.pdf]

pharmaceutics

MDPI

Supplementary Materials: Silica Coating of Ferromagnetic Iron Oxide Magnetic Nanoparticles Significantly Enhances Their Hyperthermia

Cristian Iacovita, Ionel Fizesan, Stefan Nitica, Adrian Florea, Lucian Barbu-Tudoran, Roxana Dudric, Anca Pop, Nicoleta Vedeanu, Ovidiu Crisan, Romulus Tetean, Felicia Loghin and Constantin Mihai Lu-caciu

1. Scanning Transmission Electron Microscopy analysis:

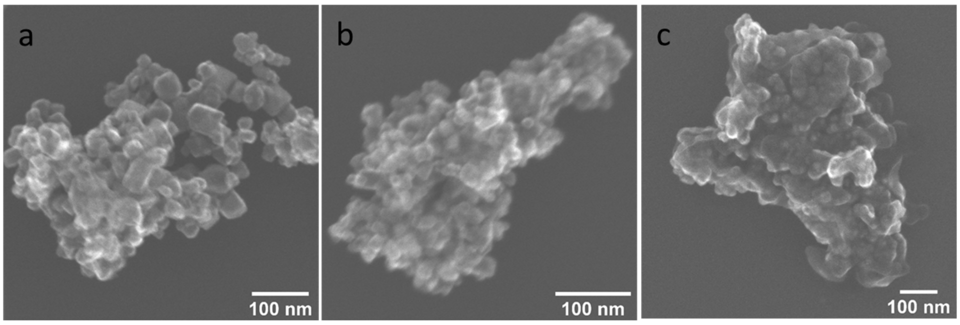

Figure S1. STEM images of silica-coated polyhedral Fe_3O_4 with different silica shell thickness (**a**) $Fe_3O_4@SiO_2$-1, (**b**) $Fe_3O_4@SiO_2$-2, and (**c**) $Fe_3O_4@SiO_2$-3.

2. X-ray Diffraction analysis:

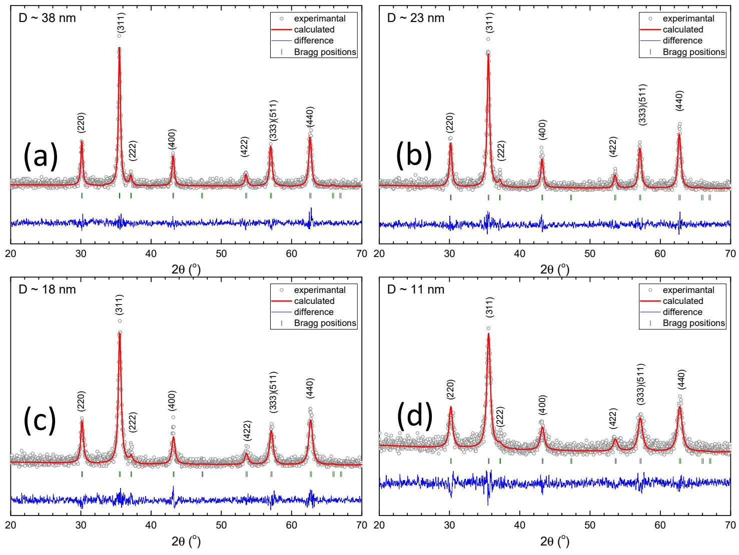

Figure S2. XRD patterns of (**a**) bare polyhedral Fe_3O_4 MNPs and sFe_3O_4 MNPs: (**b**) $Fe_3O_4@SiO_2$-1, (**c**) $Fe_3O_4@SiO_2$-2, and (**d**) $Fe_3O_4@SiO_2$-3. The average length and lattice parameter are indicated for each sample in the upper left corner of the graphs.

3. Dynamic Light Scattering analysis:

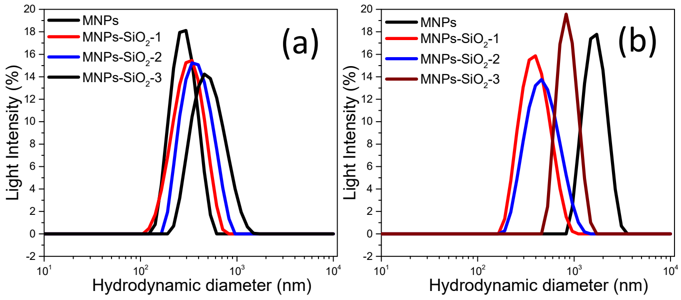

Figure S3. Hydrodynamic diameter resulted from DLS measurements of all four types of MNPs dispersed in water at concentration of (a) 0.01 mg_{MNPs}/mL and (b) 0.1 mg_{MNPs}/mL.

Table S1. Polidispersivity index values obtained from DLS data.

Samples	PDI	
	**0.01 mg_{Fe}/mL**	**0.1 mg_{Fe}/mL**
Fe_3O_4	0.273	0.228
$Fe_3O_4@SiO_2$-1	0.226	0.216
$Fe_3O_4@SiO_2$-2	0.182	0.172
$Fe_3O_4@SiO_2$-3	0.343	0.263

4. Heating curves for all types of MNPs dispersed in water at different concentrations of iron content:

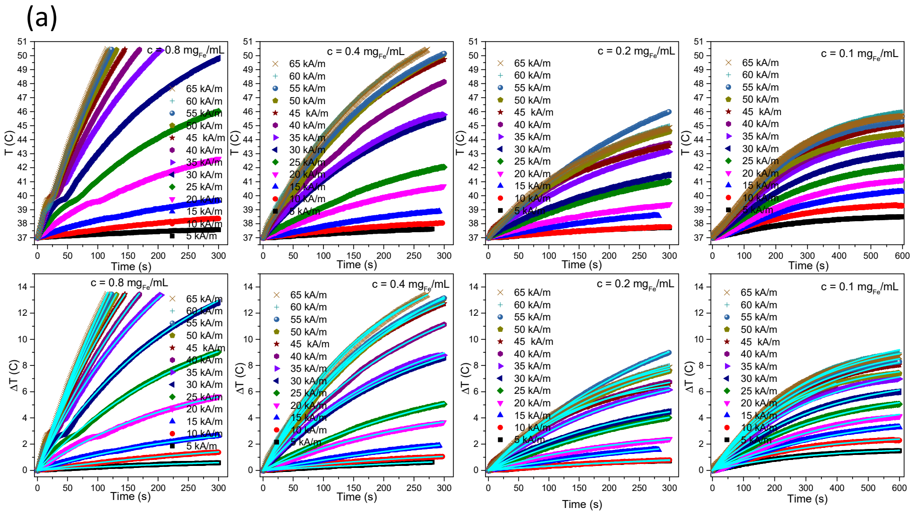

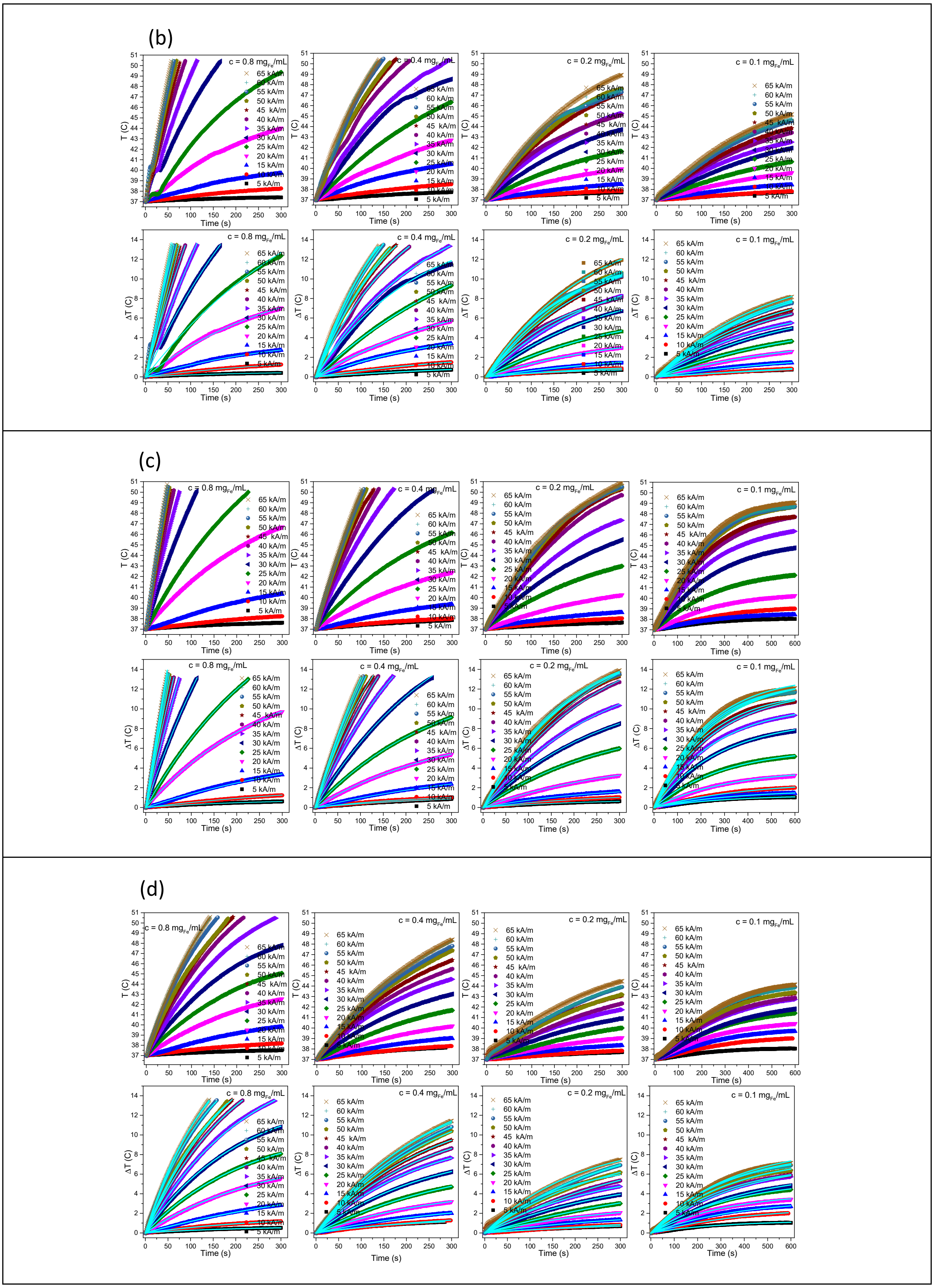

Figure S4. Groups of panels displaying the heating curves and their correspondings temperature change ΔT versus time curves fitted with Box-Lucas equation (blue curves) of (a) Fe_3O_4, (b), $Fe_3O_4@SiO_2$-1, (c) $Fe_3O_4@SiO_2$-2 and (d) $Fe_3O_4@SiO_2$-3 MNPs dispersed in water at concentrations of 0.8 mg_{Fe}/mL (first row), 0.4 mg_{Fe}/mL (second row), 0.2 mg_{Fe}/mL (third row) and 0.1 mg_{Fe}/mL (fourth row), recorded as a function of AC magnetic field amplitudes at frequency of 355 kHz.

5. The method of Specific Absorption Rate (SAR) determination:

The specific absorption rate (SAR) is defined as the heat released from a suspension of MNPs in unit time reported to the mass of iron content. It was used to quantify the heat performance of MNPs. For reliable determination of SAR, the temperature change ΔT versus time curves - where $\Delta T = T(t) - T_0$; T(t) is the temperature at time t and $T_0 = 37$ °C -, have been fitted with the Box-Lucas equation:

$$\Delta T = \frac{S_m}{k}\left(1 - e^{-k(t-t_0)}\right) \qquad (1)$$

where the fitting parameters $\boldsymbol{S_m}$ and $\boldsymbol{k}$ are the initial slope of the heating curve and the constant describing the cooling rate, respectively. Thus, SAR can be calculated as:

$$\text{SAR} = \frac{c\, m\, S_m}{m_{Fe}} \qquad (2)$$

where c is the specific heat of the colloid (in our case was approximated with the specific heat of water: $c = 4186.8 \frac{\text{J}}{\text{kg K}}$ the MNPs contribution to the specific heat being negligible), $\text{m} = \rho\text{V}$ is the mass of colloid, taken as the product between the density ($\rho_{water} = 0{,}997 \frac{\text{g}}{cm^3}$)and the volume. The iron concentration of samples was determined using the thiocyanate assay described in the section below. Before each measurement, the samples have been sonicated for 15 seconds to assure a good colloidal dispersion over the entire aqueous volume. Each SAR value is a mean of three measurements realized on three different samples.

6. Iron concentration determination:

The iron content of samples was measured using the thiocyanate assay. Silica-coated MNPs from 1 mL of a colloidal suspension were magnetically separated and further suspended in 10 mL of HCl 12% solution for digestion at 80°C for at least 4 h. The incubation was followed by centrifugation at 12000 g for 10 mins and the supernatants were collected for Fe^{3+} quantification. All iron species were oxidized to Fe^{3+} by incubating 50 μL of the supernatant with 50 μL of 1% ammonium persulfate for 30 mins. the colored Fe^{3+}-thiocyanate compound was obtained by adding 100 μL of 0.1 M potassium thiocyanate and the absorbance was measured at a λ = 490 nm using the Synergy 2 Multi-Mode Microplate Reader. The Fe^{3+} content of NPs was calculated from a Fe^{3+} standard curve with concentrations ranging between 2.5 - 140 μg/mL (SI). The possible interference of the silica coating in the quantification of Fe^{3+} was also evaluated. The digested sample of a 4 mg_{MNPs}/mL suspension of silica-coated MNPs was spiked with similar concentrations of Fe^{3+} as the ones used for the above-mentioned calibration curve and the calibration curves were compared after a blank subtraction in case of the spiked calibration curve.

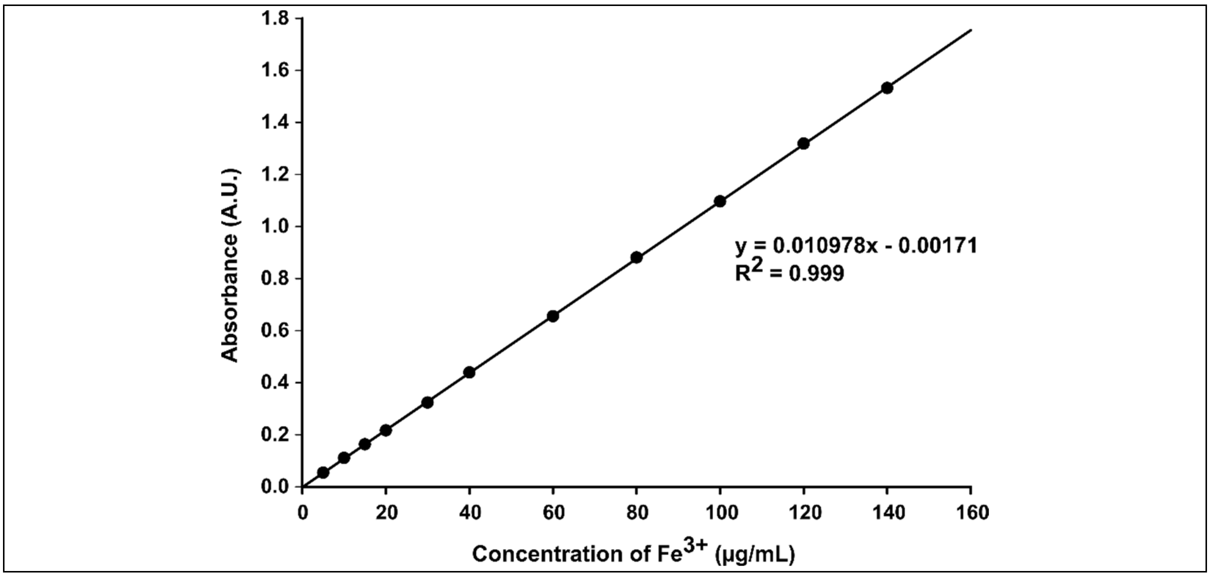

Figure S5. The absorbance of six standard Fe^{3+} colloidal solutions as a function of Fe^{3+} concentration was measured at a λ = 490 nm. The values are expressed as mean ± SD of three replicates. The black line represents a linear regression of the experimental values.

7. The logistic function used in fitting the the SAR values as a function of H:

The sigmoidal evolution of our experimental SAR data with H was well fitted (R^2 > 0.999) phenomenologically with a simple logistic function:

$$SAR = SAR_{max}\frac{\left(\frac{H}{H_{cHyp}}\right)^{n}*\propto}{1+\left(\frac{H}{H_{cHyp}}\right)^{n}*\propto} \quad \text{(S1)}$$

with:

$$\propto = \frac{n+1}{n-1} \quad \text{(S2)}$$

where SAR_{max} - the saturation value of the SAR, H_{cHyp} - the hyperthermia coercive field, the value of the H for which the function presents the highest slope or the H at which the first derivative of SAR against H presents a maximum, and the exponent n – which indicates how steep is the dependence of SAR on H. The values of these parameters for all four types of MNPs at each iron concentration are provided in Table SI1.

Table S2. Fitting parameters of SAR evolution with H.

Sample	Conditions	c (mg_{Fe}/mL)	SAR_{max} (W/g_{Fe})	H_{cHyp} (kA/m)	Power Coefficient n
Fe_3O_4	Water	0.8	770 ±11	23.67 ±0.4	3.35 ±0.12
		0.4	1070 ±25	24.05 ±0.7	3.05 ±0.13
		0.2	1190 ±28	23.81 ±0.7	2.84 ±0.12
		0.1	1305 ±34	19.50 ±0.8	2.43 ±0.12
$Fe_3O_4@SiO_2$-1	Water	0.8	1345 ±45	29.33 ±0.9	3.41 ±0.21
		0.4	1770 ±90	26.97 ±1.6	2.77 ±0.21
		0.2	1720 ±37	25.65 ±0.6	3.07 ±0.13
		0.1	1950 ±29	23.47 ±0.4	2.87 ±0.08
$Fe_3O_4@SiO_2$-2	Water	0.8	1855 ±37	28.65 ±0.5	3.77 ±0.16

		0.4	1960 ±20	27.49 ±0.3	3.68 ±0.10
		0.2	2140 ±36	26.39 ±0.5	3.49 ±0.13
		0.1	2380 ±37	25.47 ±0.4	3.44 ±0.13
Fe_3O_4@SiO_2-3	Water	0.8	615 ±10	23.15 ±0.5	3.16 ±0.11
		0.4	760 ±20	23.59 ±0.7	2.82 ±0.12
		0.2	975 ±26	24.29 ±0.9	2.43 ±0.09
		0.1	960 ±20	22.41 ±0.6	2.64 ±0.11

8. SAR dependence on H for the samples dispersed in solid PEG 8K

We performed MH experiments in polyethylene glycol 8000 which is solid at the hyperthermia temperature, the MNPs being dispersed in the solvent heated at 80 ^{0}C, under ultrasonication, and allowed to cool down to room temperature. The results presented in the figure below show clearly that the sample Fe_3O_4@SiO_2-2 has the best heating properties (panel a).

For all samples, we noticed a decrease in the SAR values, due to the solid matrix which blocks the physical rotation of the particles and thus the Brown relaxation mechanism. The relative MH performances are preserved within the samples and they are in the same order of the stability we mentioned above. Also one can notice that the decrease in the SAR is more pronounced for the same sample Fe_3O_4@SiO_2-2 (from 1800 W/g to 1200 W/g) meaning that this sample has also the largest contribution by Brown relaxation to the heating, being the most mobile one.

Moreover, when the concentration of the sample Fe_3O_4@SiO_2-2 was changed, no significant change in SAR was noticed (panel b). This might be explained by the fact that the NPs are well dispersed within the matrix and during MH they cannot aggregate, increase their dipolar interactions and thus decrease the SAR, a phenomenon that depends strongly on the concentration.

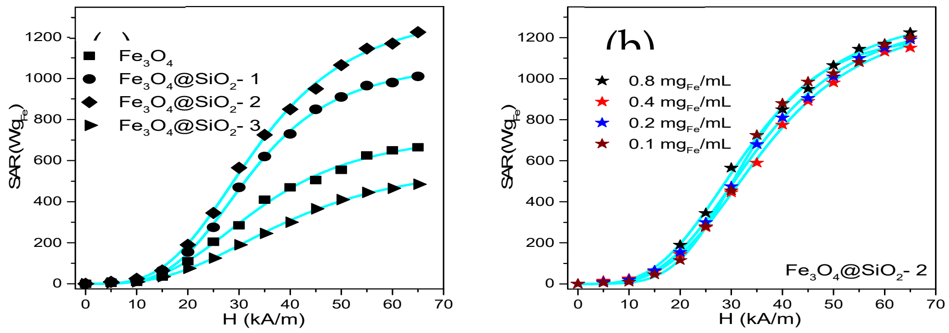

Figure S6. SAR dependence on the strength of magnetic field H for the 4 samples dispersed in solid PEG 8 K at a concentration of 0.8 mg/mL (panel a) and the same dependence for the sample Fe3O4@SiO2-2 at 4 different concentrations (panel b).

9. Optical and biochemical interference of Fe_3O_4@SiO_2-2 MNPs with Alamar Blue and Neutral Red assays:

Before the evaluation of the Fe_3O_4@SiO_2-2 MNPs cytotoxicity, their optical and biochemical interferences with the viability assays were evaluated. In the case of Alamar Blue (AB), the ability of the Fe_3O_4@SiO_2-2 MNPs to reduce resazurin to the measured fluorescent compound, resorufin, or to adsorb or re-oxidize resorufin to resazurin was evaluated by incubating for 3 h different concentrations of MNPs with resazurin (Sigma-Aldrich, Steinheim, Germany) or resorufin (prepared extempore by autoclaving a resazurin solution). After the incubation period, the suspension mixture was centrifuged to remove MNPs, and the fluorescent signal of the supernatant was measured using a Synergy 2 Multi-Mode Microplate Reader (BioTek® Instruments Inc., Winooski, USA). Similarly, the ability of Fe_3O_4@SiO_2-2 MNPs to interfere with the Neutral Red (NR) assay, by adsorption of the dye, was evaluated. Optical interference

assays were conducted by measuring the emitted fluorescence of resorufin and neutral dye solution in an extempore prepared mixture with different concentrations of MNPs.

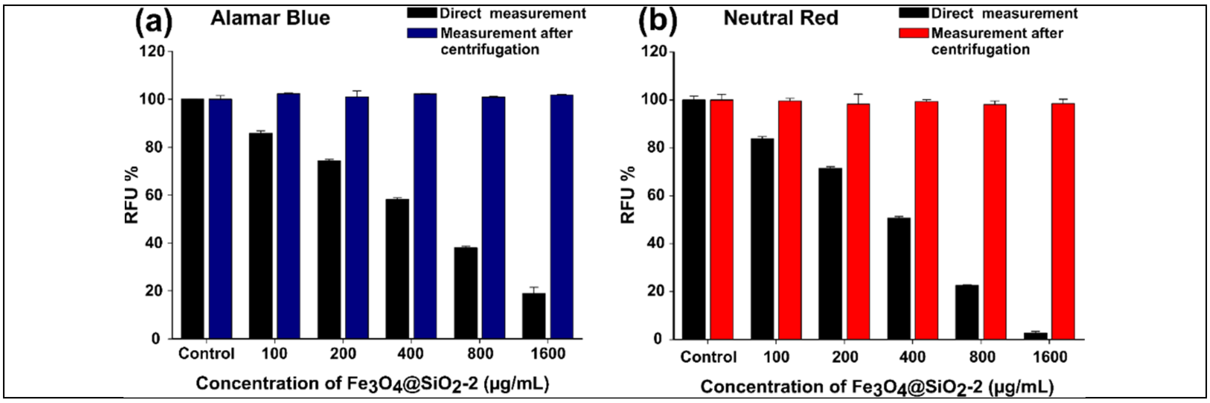

Figure S7. Optical interference of Fe_3O_4@SiO_2-2 MNPs with Alamar Blue assay (**a**) and Neutral Red assay (**b**). Different quantities of Fe_3O_4@SiO_2-2 MNPs were extempore mixed with resorufin and neutral red and the fluorescence was measured directly or after a centrifugation step. The values are expressed as mean ± SD of three replicates. Data were expressed as relative values to the negative control.

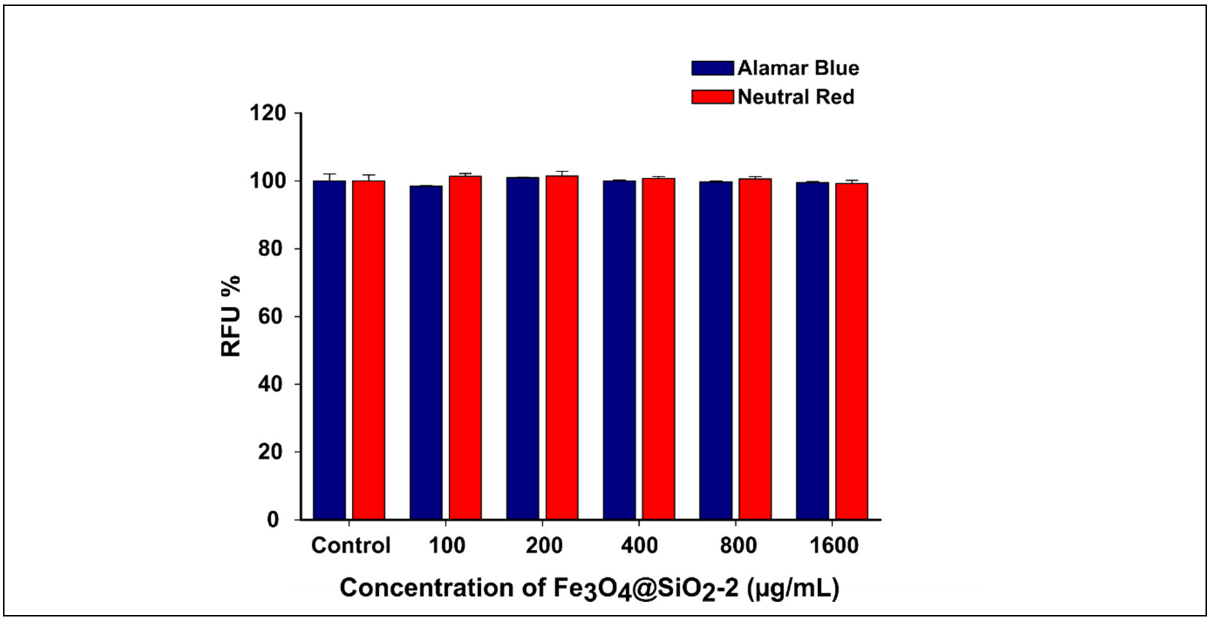

Figure S8. Biochemical interference of Fe_3O_4@SiO_2-2 MNPs with Alamar Blue assay and Neutral Red assay. Different quantities of Fe_3O_4@SiO_2-2 MNPs were incubated with resorufin and neutral red for 3 hours. After the incubation, all samples were centrifuged and the fluorescence was measured. The values are expressed as mean ± SD of three replicates. Data were expressed as relative values to the negative control.

10. Cellular uptake of MNPs:

The cells suspended in 2 mL of cell medium were seeded in 6-well plates and afterward exposed to 1 mL of cell medium containing MNPs to reach a concentration of 62.5 to 7.81 $\mu g_{MNPs}/cm^2$. Based on +the thiocyanate assay the intracellular iron content was determined upon 24 h of incubation. For MH treatment, two equally aliquots of 1.5 mL each were separated, the cells were gently centrifuged and 1.3 mL of cell culture media was removed from each aliquot.

Consequently, the iron concentration of the samples, containing MNPs loaded cancer cells, exposed to AFM (the last columns from table S3) has been calculated by dividing half of the internalized amount of Fe^{3+} ions to 0.2 mL (200 μL).

Table S3. Amount of Fe^{3+} internalized in cells relevant for in-vitro cytotoxicity assays and Fe^{3+} concentration of samples used in *in vitro* magnetic hyperthermia.

Cell	Exposed dose of MNPs (μg /cm²)	Exposed amount of MNPs (μg)	Internalized amount of Fe^{3+} ions (μg)	In vitro magnetic hyperthermia concentrations (mg_{Fe} / mL)
BJ	62.5	625	119.29	0.29823
	31.25	312.5	88.261	0.22065
	15.62	156.2	54.83	0.13708
	7.81	78.1	28.303	0.07076
A549	62.5	625	234.595	0.58649
	31.25	312.5	134.654	0.33664
	15.62	156.2	69.184	0.17296
	7.81	78.1	34.115	0.08529
A375	62.5	625	201.614	0.50404
	31.25	312.5	119.495	0.29874
	15.62	156.2	57.639	0.1441
	7.81	78.1	31.794	0.07949

11. SEM and TEM examination of A549, A375, and BJ cells:

The lung adenocarcinoma A549 cells were observed at SEM examination as adherent, flat cells (of about 30-40 μm across), almost confluent, showing thin and short filopodia present in high number as extracellular extensions of plasma membrane – used for probing the environment and for cell-to-cell communication; the more prominent, central region of the cells corresponding to the intracellular location of nucleus (Fig. S8a). The cellular inner organization is revealed by TEM: the cells contained large euchromatic nuclei, with an irregular shape, and large nucleoli; in the cytoplasm, rare oval mitochondria, several short profiles of endoplasmic reticulum and many free ribosomes were identified. The thin filopodia were also seen in the section at the cellular periphery (Fig. S8b).

The melanoma A375 cells appeared in SEM as adherent, more or less triangular cells (of about 60-70 μm in length – but also cells of more than 90 μm were found), with large and thick prolongations (of about 10 μm in diameter). These large prolongations were covered with thin filopodia, and rare lamellipodia (thin and flat cellular extensions) were found as well, which in turn generated more filopodia (Fig. S8c). The inner structure of these cells was more complex: apart from the large and euchromatic nuclei, they contained more organelles dispersed within the cytoplasm: many round-oval mitochondria, abundant endoplasmic reticulum, electron-lucent vacuoles of various sizes, and numerous free ribosomes (Fig. S8d).

The BJ cells were also adherent to the coverslips; they were long (of about 120 μm or more in length) stretched and branched cells, some of them displaying even triangular shapes. At their surface filopodia were present in high numbers as very long extensions, apparently interconnecting the neighboring cells (Fig. S8e). These cells had large, round, and predominantly euchromatic nuclei, and numerous cytoplasmic organelles: small round or oval mitochondria, endoplasmic reticulum, autophagosomes, and rare small vacuoles; also many free ribosomes were visible (Fig. S8f).

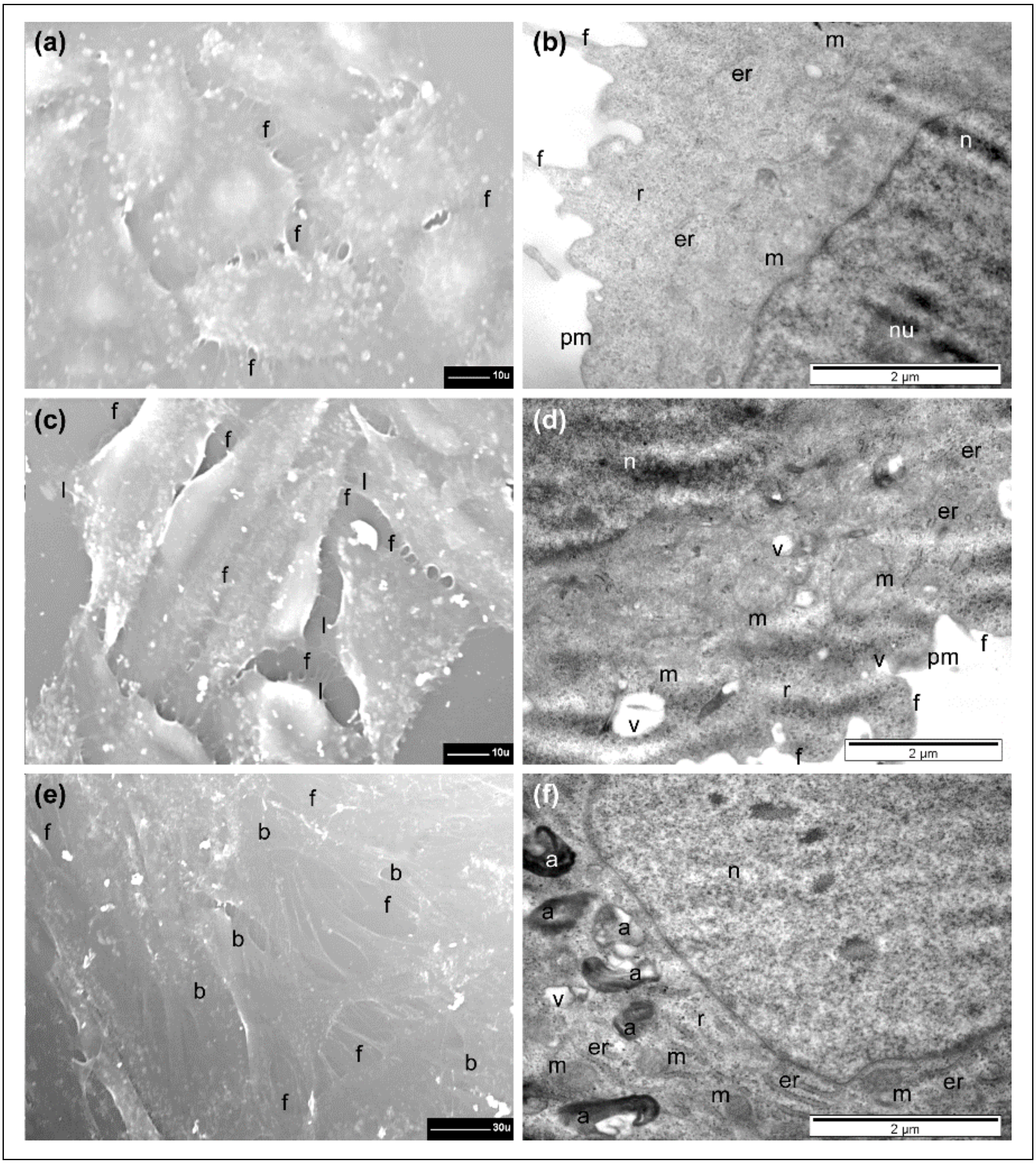

Figure S9. SEM and TEM images of (**a**) and (**b**) A549; (**c**) and (**d**) A375; (**e**) and (**f**) BJ, revealing their external appearance and internal structure. The significance of letters is: f = filopodia, l = lamellipodia, r = ribosome, er = endoplasmic reticulum, pm = plasma membrane, m = mitochondria, n = nucleus, nu = nucleolus, v = vacuole, a = autophagosome and b = branche.

12. SEM and TEM examination of A549, A375, and BJ cells incubated with $Fe_3O_4@SiO_2$-2 MNPs:

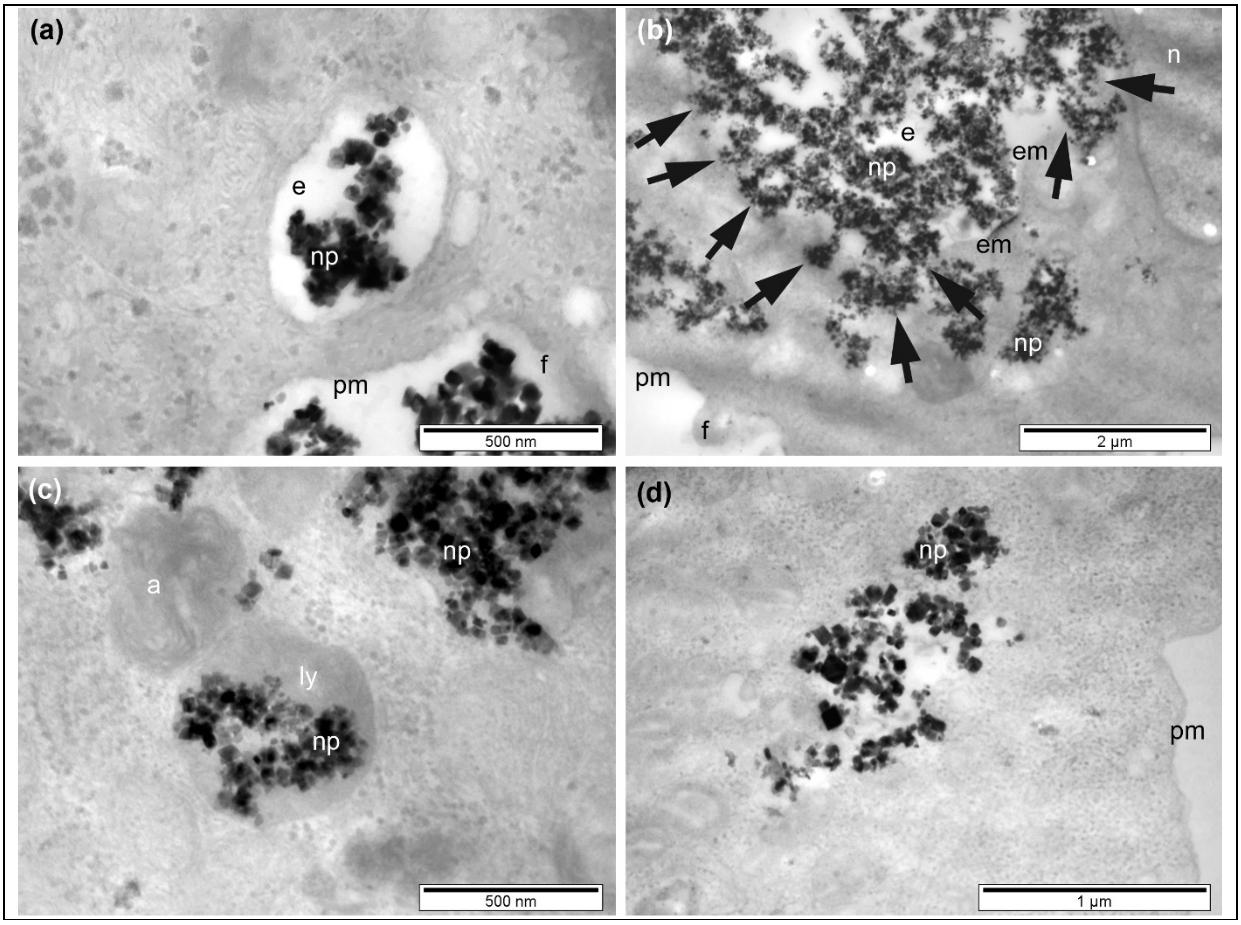

Figure S10. Relevant TEM images of BJ (a) A579 b) BJ c) and A375 cells (d) incubated with $Fe_3O_4@SiO_2$-2 MNPs for 24 h in a dose of 31.25 μg/cm^2 to show the intracellular presence of nanoparticles in endosome (a); large endosomes with partially disrupted membranes and cytosol (b) lysosome and cytosol (c) and dispersed into the cytosol (d). The significance of letters is: a = autophagosome, e = endosome,em= endosomal membrane f = filopodia, np = nanoparticles, ly = lysosome,n= nucleus and pm = plasma membrane. Arrows indicate regions where the endosomal membrane is disrupted with releasing the NPs into the cytosol.

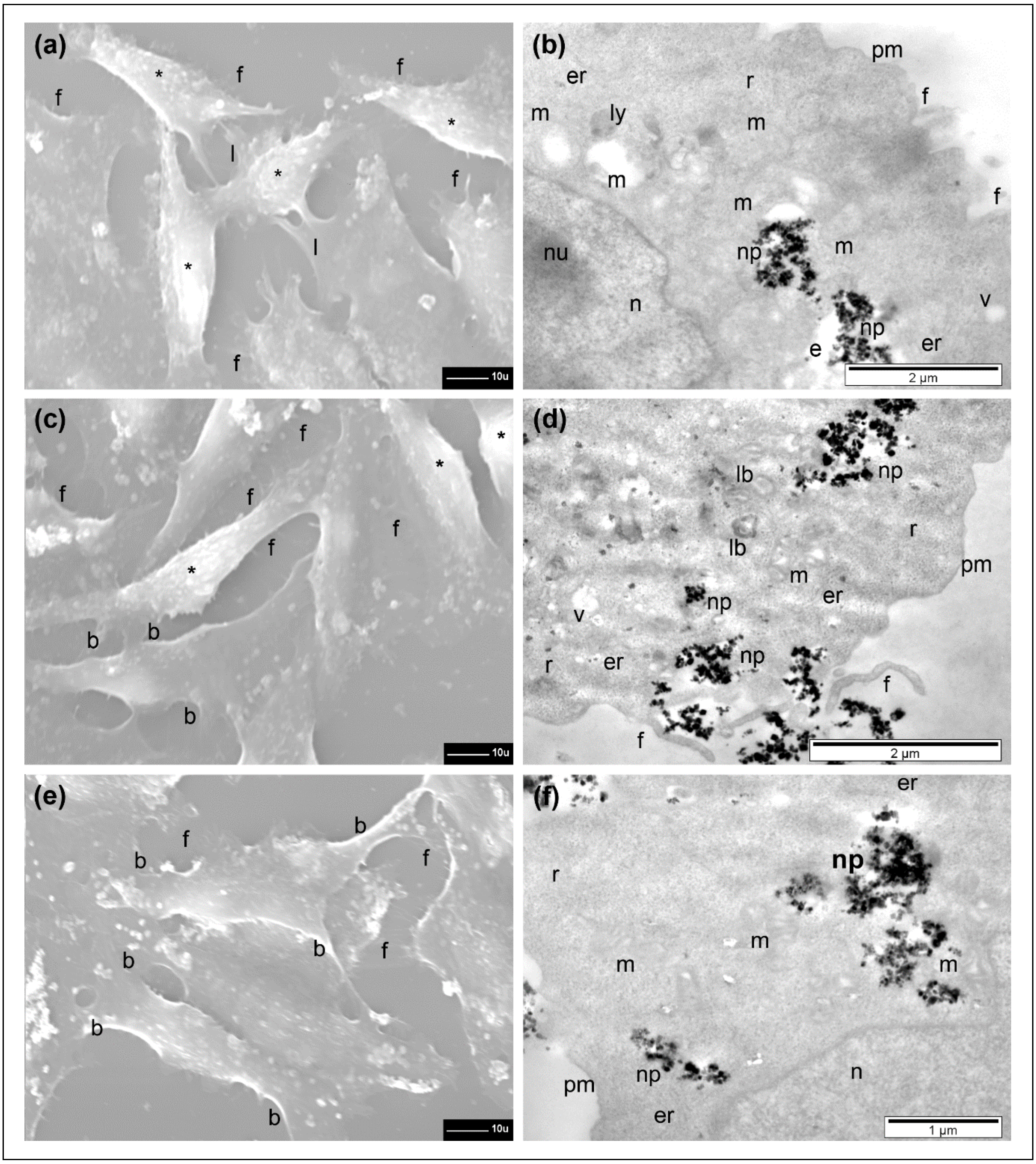

Figure S11. SEM and TEM images of A375 cells containing $Fe_3O_4@SiO_2$-2 MNPs after 24 h incubation time in a dose of (**a**) and (**b**) 15.62 µg/cm^2, (**c**) and (**d**) 31.25 µg/cm^2 and (**e**) and (**f**) 62.5 µg/cm^2. The significance of letters is: f = filopodia, l = lamelipodia, b = branche, np = nanoparticles, er = endoplasmic reticulum, r = ribosome, m = mitochondria, pm = plasma membrane, v = vacuole, lb = lamellar bodies, n = nucleus, nu = nucleolus, e = endosome and ly = lyosome. The asterisks indicate contracted cells.

13. Heating curves of MNPs $Fe_3O_4@SiO_2$-2 MNPs internalized in BJ, A549, and A375 cells at different concentrations:

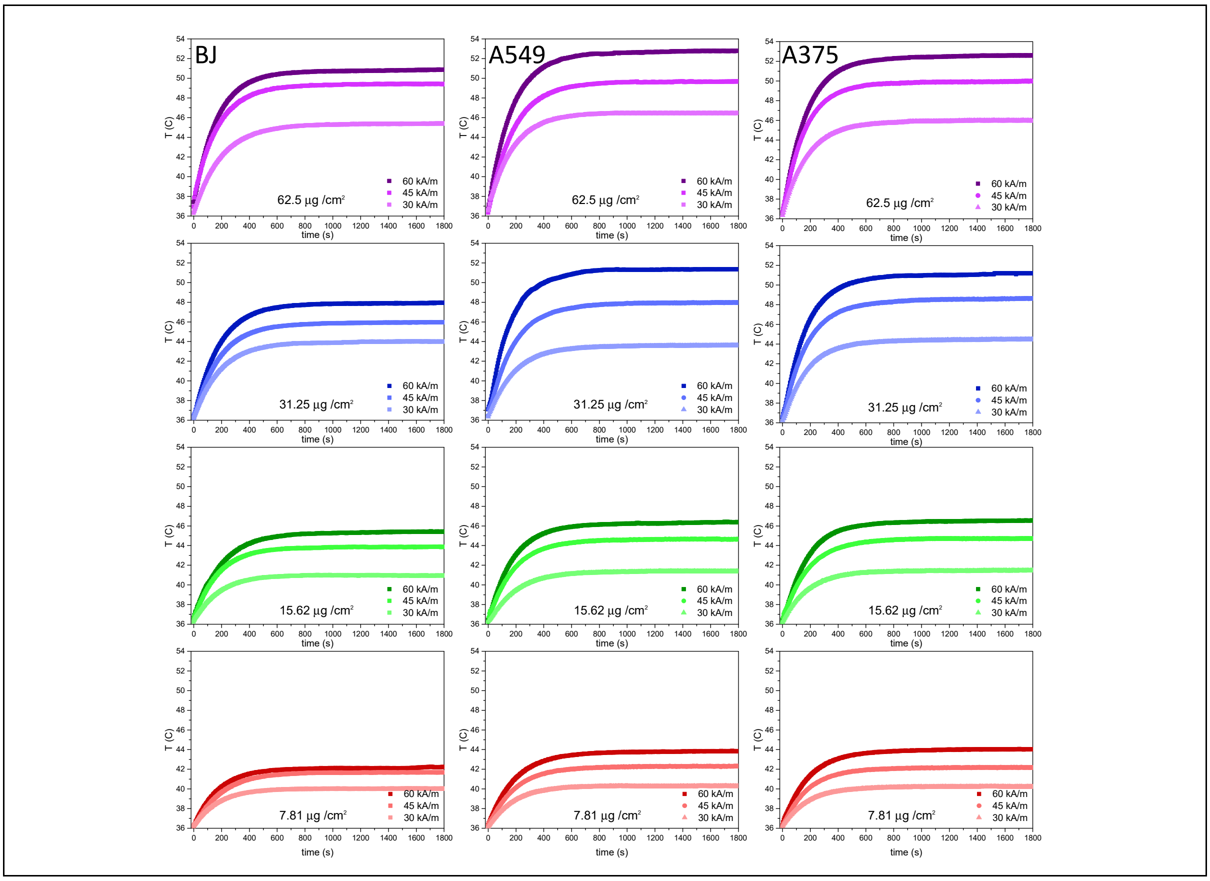

Figure S12. Groups of panels displaying the heating curves of $Fe_3O_4@SiO_2$-2 MNPs internalized in BJ (left column), A549 (middle column), and A375 (right column) cells at different concentrations, recorded at three different H values of 30 kA/m, 45 kA/m, and 60 kA/m and a constant frequency of 355 kHz.

14. Saturation temperatures reached during in vitro MH experiments:

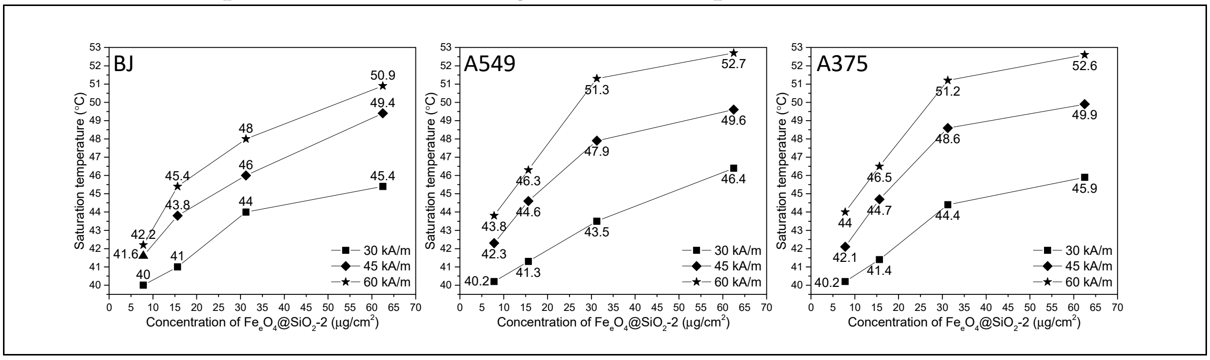

Figure S13. Saturation temperatures of $Fe_3O_4@SiO_2$-2 MNPs internalized in BJ (left panel), A549 (middle panel), and A375 (right panel) cells at different concentrations reached upon 30 minutes exposure to AC magnetic field of three H values (30, 45, and 60 kA/m) and at a frequency of 355 kHz.

15. SEM and TEM examination of A375 cells incubated with $Fe_3O_4@SiO_2$-2 MNPs and exposed for 30 min. to an AMF of 30kA/m, 355 kHz:

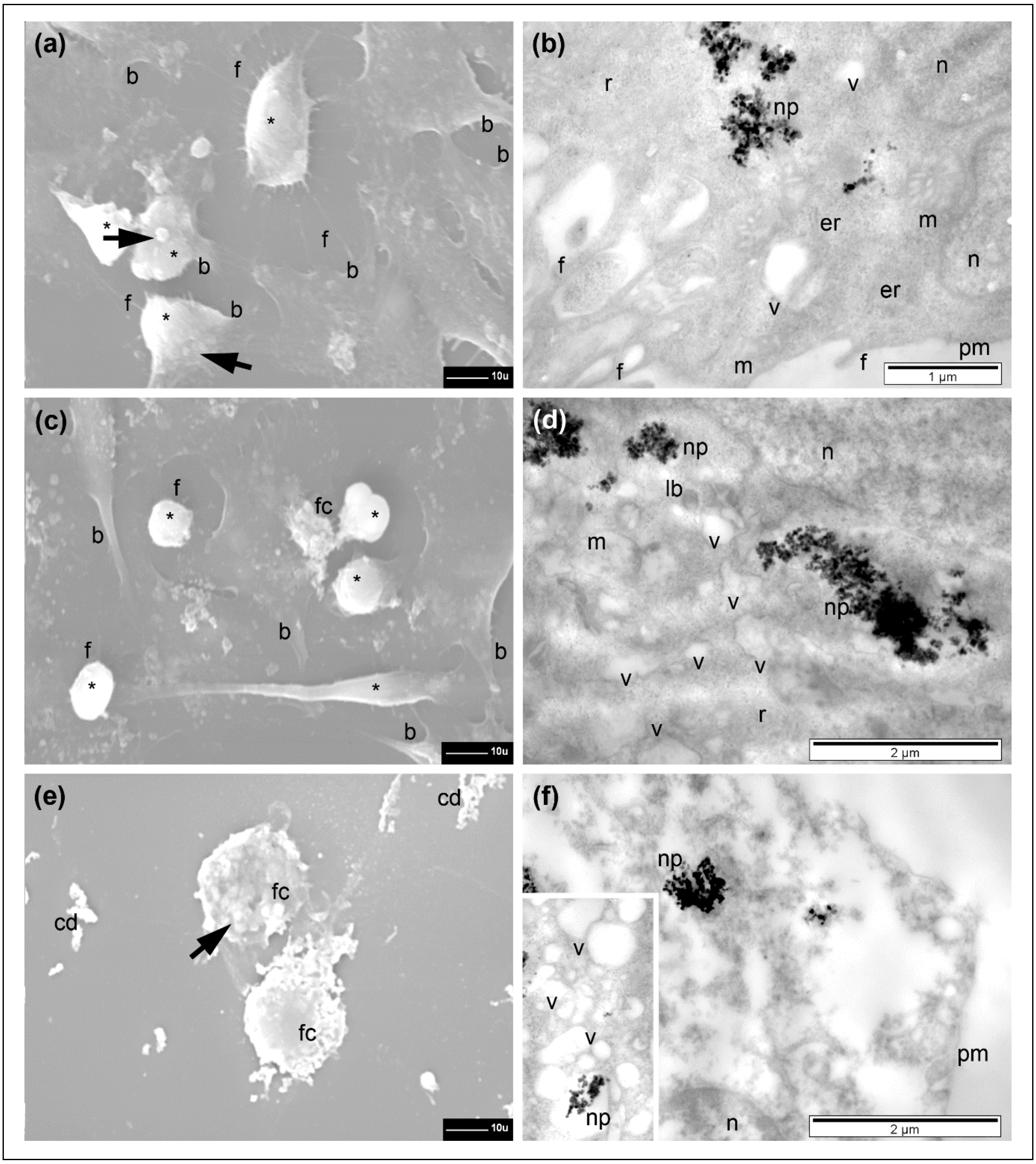

Figure S14. SEM and TEM images of A375 cells incubated with $Fe_3O_4@SiO_2$-2 MNPs for 24 h in a dose of (**a**) and (**b**) 15.62 µg/cm², (**c**) and (**d**) 31.25 µg/cm² and (**e**) and (**f**) 62.5 µg/cm² and exposed for 30 min to an AFM of 30kA/m, 355 kHz. The significance of letters is: f = filopodia, b = branche, np = nanoparticles, v = vacuole, m = mitochondria, r = ribosome, n = nucleus, er = endoplasmic reticulum, pm = plasma membrane, lb = lamellar bodies, cd = celular debris and fc = faulted cell.The inset (**f**, left) show large vacuoles and nanoparticles. The asterisks indicate contracted cells. The black arrows indicate membrane blebs.

16.

Table S4. Fitting parameters of cytotoxicity data upon intracellular hyperthermia with eq.1 from the main paper. T_0 the temperature for 50 % viability.

	Alamar Blue		Neutral Red	
**Cell Type**	**T_0**	**dT**	**T_0**	**dT**
**BJ**	44.2+/-0.2	1.2+/-0.2	47.3+/-0.4	1.5+/-0.3
**A459**	43.6+/-0.2	0.8+/-0.2	45.2+/-0.2	0.6+/-0.1
**A375**	43.6+/-0.2	1.0+/-0.2	44.6+/-0.2	0.7+/-0.2

.
